# Valorization of Olive Pomace as a Source of Phenolic Compounds: Extraction Technologies, Analytical Characterization, Biological Activities, and Food Applications

**DOI:** 10.3390/foods15162943

**Published:** 2026-08-21

**Authors:** Leyla Sanhueza, Sonia Morante-Zarcero, Isabel Sierra

**Affiliations:** 1 Departamento de Tecnología Química y Ambiental, Escuela Superior de Ciencias Experimentales y Tecnología (ESCET), Universidad Rey Juan Carlos, C/Tulipán s/n, 28933 Móstoles, Spain; leyla.sanhueza.kaba@urjc.es; 2 Instituto de Investigación de Tecnologías para la Sostenibilidad, Universidad Rey Juan Carlos, C/Tulipán s/n, 28933 Móstoles, Spain

**Keywords:** chromatographic analysis, spectrophotometric assays, circular economy, agro-industrial by-products, antioxidant activity, olive pomace, phenolic compounds, green extraction

## Abstract

The valorization of agro-industrial by-products has gained increasing attention as a sustainable strategy to support circular economy principles and reduce environmental impacts. Spain, the world’s largest olive oil producer, generates substantial amounts of olive pomace (OP), which can represent up to 80% of the processed olive mass. Due to their hydrophilic nature, approximately 98% of olive phenolic compounds remain in OP after oil extraction, making this by-product a valuable source of bioactive compounds with antioxidant, anti-inflammatory, antimicrobial, and cardioprotective properties. Numerous extraction strategies have been investigated to maximize phenolic recovery while reducing processing costs and environmental impacts. Conventional solvent-based techniques, such as solid–liquid extraction (SLE) and liquid–liquid extraction (LLE), remain widely used, while greener approaches, including microwave-, ultrasound-, supercritical fluid-, pressurized liquid-, and high-pressure-assisted extraction, among others, have gained increasing attention. In addition, deep eutectic solvents (DES) have been applied either alone or in combination with green extraction technologies to enhance extraction efficiency. This review provides a comprehensive overview of extraction techniques for OP valorization and the analytical methodologies used to characterize OP extracts, including spectrophotometric and chromatographic approaches. The biological activities of OP-derived phenolics and their food applications are also discussed, highlighting their valorization potential.

## 1. Introduction

Food waste generation has become a major global concern. The publication of reports by the Food and Agriculture Organization of the United Nations (FAO) on global food loss and waste has further emphasized this issue, highlighting the significant environmental implications of reducing food waste. According to the most recent FAO report, up to 1.3 billion tons of food are lost or wasted annually worldwide, accounting for approximately 30% of total global food production. Therefore, the valorization of food waste is a key strategy for enhancing environmental and economic sustainability throughout the global food supply chain [1,2,3,4].

The European Union (EU) has established a legislative framework to promote the circular economy, which includes the agri-food sector. Within this framework, the Circular Economy Action Plan (CEAP) articulates the EU’s commitment to reducing agri-food waste across its Member States. In Spain, this policy is reinforced by Law 1/2025 of 1 April on the prevention of food loss and waste, together with Law 7/2022 of 8 April on waste and contaminated soils for a circular economy. Collectively, these legal instruments seek to minimize food waste through the implementation of mechanisms designed to reduce losses throughout the food supply chain. A key strategic approach to achieving this objective is the valorization of food by-products via their transformation into new products, thereby fostering the principles of the circular economy [5,6,7]. Beyond the European Union, which has established a comprehensive legislative framework through the Circular Economy Action Plan, several countries have implemented regulatory instruments and national strategies to foster resource efficiency and waste valorization. Examples include China’s Circular Economy Promotion Law, Japan’s Food Recycling Act, and national circular economy roadmaps adopted in countries such as South Korea, Canada, Australia, Chile, and Colombia, many of which explicitly recognize the agri-food sector as a key area for circularity and biomass valorization [5,8,9,10,11,12,13].

Approximately 20% of food industry waste is generated by the processing of oilseeds, dairy products, and meat, a category that includes olives [14,15]. One promising approach for the valorization of these residues is the extraction of bioactive compounds from plant-derived by-products. Many of these by-products are rich in antioxidants, including phenolic compounds (such as flavonoids and phenolic acids) and carotenoids (e.g., β-carotene and lycopene). These compounds have demonstrated potential health benefits; for example, phenolic compounds act as free radical scavengers, thereby mitigating the harmful effects of reactive oxygen species generated during aerobic metabolism and reducing oxidative stress in the human body [15,16].

The cultivation of *Olea europaea* L., commonly known as the olive tree, represents one of the oldest and most economically and culturally significant agricultural activities in the Mediterranean basin. This species, belonging to the Oleaceae family, is the only member of the family that produces fruits suitable for olive oil extraction. Spain is the world’s leading producer and exporter of olive oil and possesses the largest olive cultivation area worldwide. During the 2024/2025 crop season, Spain accounted for approximately 40% of global olive oil production. Olive oil production in Spain during the period from October 2024 to May 2025 reached approximately 1.4 million tonnes, generating between 4 and 6 million tonnes of olive pomace (OP) as an industrial by-product [17,18,19]. Following the oil extraction process, OP emerges as one of the most important by-products generated. This residue is initially characterized by a pinkish, mud-like appearance that rapidly oxidizes and turns brown. It is a heterogeneous material composed of fragments of olive skin and pulp, stone pieces, seeds, residual oil, and water [19]. The OP that can represent up to 80% of the total mass of processed olives, is composed of approximately 60–80% water, 15–30% lipids, 3–6% carbohydrates, 1–3% proteins and 1.5–2% tannins and other substances such as organic acids, pectins, phenolic compounds and minerals. It has been observed that most of the phenolic compounds originally present in olives remain in this by-product generated during oil extraction, where their concentrations are significantly higher than those found in olive oil. Consequently, and considering the large quantities generated, there is growing interest in the valorization of OP. The recovery and utilization of these bioactive compounds represent a promising opportunity to transform this agro-industrial residue into a valuable raw material for applications in the food industry [19,20,21].

**Figure 1:**
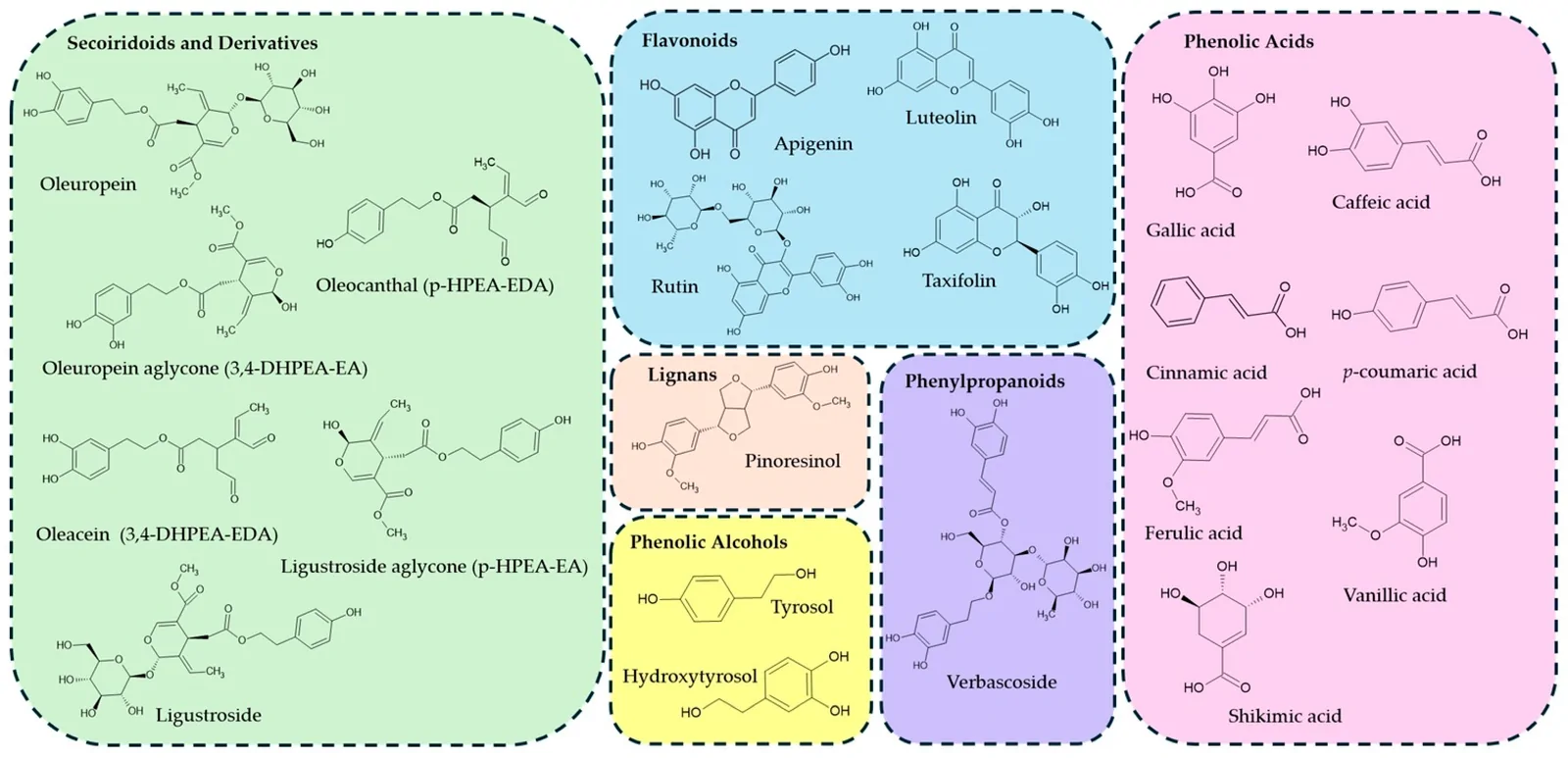
Classification and chemical structures of the main phenolic compounds identified in OP.

Among the phenolic compounds present in olive residues are hydroxytyrosol (HYT) and tyrosol (TYR) and their derivatives; iridoid and secoiridoid precursors and derivatives, including oleuropein (OLE), OLE aglycone, ligustroside, and their derivatives; phenylpropanoids such as verbascoside (VERB) and its derivatives; flavonoids, including luteolin (LUT), apigenin, rutin (RUT), taxifolin, and their derivatives; lignans, such as pinoresinol and its derivatives; and phenolic acids, including gallic acid (GAL), caffeic acid (CAFF), cinnamic acid, p-coumaric acid, ferulic acid (FER), vanillic acid (VAN), and shikimic acid (Figure 1). Although OP contains a wide variety of bioactive compounds, several studies indicate that HYT, TYR, and OLE are the predominant constituents, contributing significantly to the biological activities reported for OP [22,23,24]. HYT has been attributed a wide range of biological activities, including antimicrobial, antithrombotic, antitumor, chemoprotective, skin-bleaching, antioxidant, anti-inflammatory, antiatherogenic, and cardioprotective effects, among others, owing to these beneficial properties, the European Food Safety Authority (EFSA) has approved health claims associated with HYT consumption [22,25,26,27]. In the case of OLE, numerous in vivo studies have demonstrated its diverse biological properties, including antioxidant, anti-inflammatory, antiatherogenic, cardioprotective, antihypertensive, hypoglycemic, antimicrobial, antiviral, cytostatic, molluscicidal, and endocrine activities. TYR, on the other hand, has demonstrated efficacy in reducing oxidative damage in muscle cells. It has been associated with beneficial effects against hypertension, atherosclerosis, coronary heart disease, heart failure, insulin resistance, and inflammation. In addition, TYR exhibits antimicrobial, antitrypanosomal, antiatherogenic, and osteopenia-preventive properties. These biological activities are closely related to its high chemical stability, which allows TYR to maintain its functionality under conditions where other phenolic compounds may undergo degradation, despite exhibiting lower antioxidant activity than many other phenols [28]. Consequently, the recovery and valorization of these phenolic compounds from OP is of considerable importance from both a functional and industrial perspective [26,29,30].

This review aims to critically analyze current extraction techniques for phenolic compounds derived from OP, encompassing both conventional methods and emerging environmentally sustainable approaches for applications in food. Furthermore, it reviews the principal analytical methods employed for the identification and quantification of these compounds and examines their association with antioxidant capacity. Finally, the review addresses their potential use as food ingredients, with particular emphasis on bioactive considerations and strategies to improve compound stability and preserve antioxidant activity. This review was conducted through an extensive and systematic search of scientific literature, focusing on articles addressing the extraction and valorization of phenolic compounds from olive by-products. The literature search employed combinations of relevant keywords and their synonyms, including “extraction”, “optimization”, “bioactivity”, “cytotoxicity”, “olive pomace”, “pomace”, “olive by-product”, “pulp”, and “olive oil residue”. The databases consulted included Scopus (Elsevier), Web of Science (Clarivate Analytics), Google Scholar, and SciFinder. The selection criteria comprised peer-reviewed publications written in English, published from 2010 onwards, indexed in Q1–Q2 journals, studies of OP (no other by-products) and relevant to the field of food science. The literature search was conducted between March 2025 and July 2026. From the selected studies, information related to extraction techniques, analytical methods, cytotoxicity assessments, and food-related applications was systematically collected and critically analyzed.

## 2. Olive Pomace Valorization Strategies

Due to the hydrophilic nature of phenolic compounds, only approximately 2% of phenolic compounds are incorporated into olive oil, whereas the remaining 98% are retained in OP [25]. Consequently, numerous conventional and green extraction strategies have been investigated to extract these bioactive compounds in a sustainable manner.

**Figure 2:**
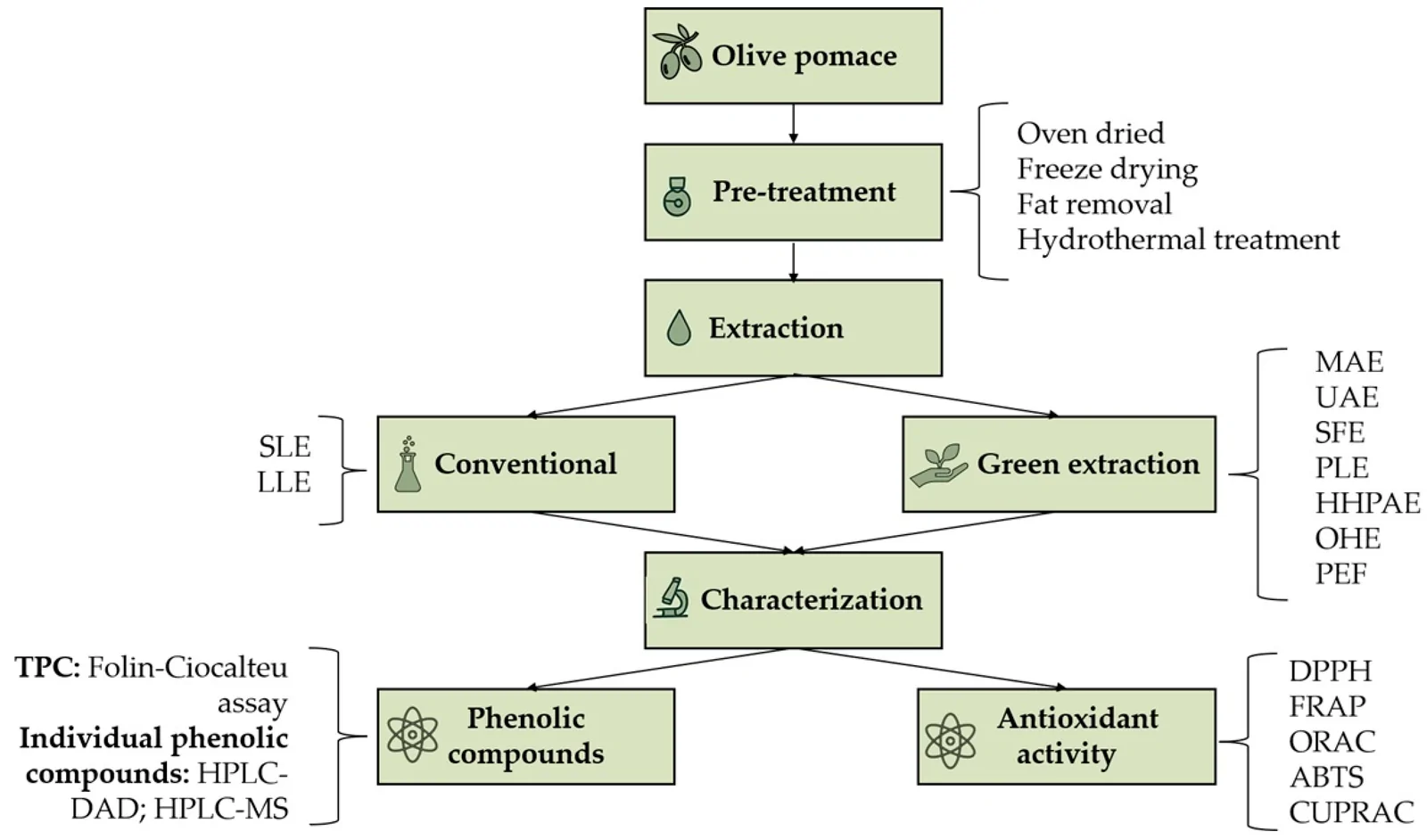
Overview of phenolic compound extraction and analytical characterization strategies in OP. SLE: solid-liquid extraction; LLE: liquid-liquid extraction; MAE: microwave-assisted extraction; UAE: ultrasound-assisted extraction; SFE: supercritical fluid extraction; PLE: pressurized liquid extraction; HHPAE: high hydrostatic pressure-assisted extraction; OHE: ohmic heating extraction; HPHT: high-pressure and high-temperature extraction; PEF: pulsed electric field-assisted extraction; DPPH: 2,2-diphenyl-1-picrylhydrazyl; FRAP: ferric reducing antioxidant power; ORAC: oxygen radical absorbance capacity; ABTS: 2,2′-azinobis (3-ethylbenzothiazoline-6-sulfonic acid); CUPRAC: cupric ion reducing antioxidant capacity.

As illustrated in Figure 2, the valorization of OP through the extraction of phenolic compounds generally involves several stages. Initially, pretreatment steps such as dehydration, defatting or hydrothermal treatments may be applied to improve extraction efficiency. Subsequently, phenolic compounds are extracted using conventional solvent-based techniques, including solid–liquid extraction (SLE) and liquid–liquid extraction (LLE), or more sustainable approaches such as microwave-assisted extraction (MAE), ultrasound-assisted extraction (UAE), supercritical fluid extraction (SFE), pressurized liquid extraction (PLE), high hydrostatic pressure-assisted extraction (HHPAE), ohmic heating extraction (OHE), combined high-pressure and high-temperature extraction (HPHT), and pulsed electric field-assisted extraction (PEF). Furthermore, deep eutectic solvents (DES) have emerged as promising alternatives to conventional solvents and have been applied in SLE as well as in combination with green extraction technologies to improve phenolic extraction.

Following extraction, the efficiency of the different extraction strategies is evaluated through the determination of total phenolic content (TPC), the identification and quantification of individual phenolic compounds by liquid chromatography, and the assessment of antioxidant activity using a range of analytical methods. Finally, the recovered compounds may be incorporated into food products or other value-added applications. The following sections describe the main pretreatment approaches, extraction technologies, and analytical methodologies employed for OP valorization.

### 2.1. Analytical Methods Used for the Quantification of Individual and Total Phenols and Antioxidant Activity Assessment in Olive Pomace Extracts

Before discussing the extraction conditions reported in the literature, it is important to describe the analytical methodologies used to characterize the resulting extracts. These methods determine how extraction efficiency is evaluated and how phenolic recovery and antioxidant activity are quantified and compared across studies.

The efficiency of the different extraction techniques is commonly evaluated using a range of analytical methods, including spectrophotometric assays for the determination of TPC, chromatographic techniques for the identification and quantification of individual phenolic compounds, and various assays for antioxidant activity assessment (Figure 2). In most cases, quantification is performed through the determination of TPC by spectrophotometry using Folin–Ciocalteu (F-C) reagent. However, some studies focus on the identification and quantification of individual phenols, likely due to the greater specificity required for such analyses. Individual phenols are typically measured using advanced chromatographic techniques such as high-performance liquid chromatography (HPLC), often coupled with detectors like diode array detection (DAD), UV-Visible (UV-Vis) detection, or mass spectrometry (MS) [31]. Additionally, antioxidant activity has been assessed using spectrophotometric assays, including 2,2-diphenyl-1-picrylhydrazyl (DPPH), Ferric Reducing Antioxidant Power (FRAP), Oxygen Radical Absorbance Capacity (ORAC), 2,2′-azinobis (3-ethylbenzothiazoline-6-sulfonic acid) (ABTS) and Cupric Ion Reducing Antioxidant Capacity (CUPRAC) [32]. The following sections describe the analytical conditions used for phenol quantification, the associated antioxidant activity assays, and the techniques used for individual compound analysis. Together, these approaches enable the evaluation of extraction efficiency and facilitate comparisons among studies.

#### 2.1.1. Spectrophotometric Methods for Total Phenolic Content Assessment

The F-C assay is one of the most widely used methods for the determination of TPC due to its simplicity, rapidity, and low cost. The method is based on the measurement of the reducing capacity of a sample and has been extensively applied to the analysis of phenolic compounds in a wide range of food matrices. This reagent contains a mixture of phosphomolybdic and phosphotungstic acids complexes that are reduced under alkaline conditions, producing a blue chromophore with a maximum absorbance at 750–765 nm. The reaction involves the transfer of electrons from phenolic compounds to the reagent and is mainly associated with the formation of Mo(V) complexes. However, the F-C assay is not entirely specific to phenolic compounds, as it also responds to other reducing substances. These include sugars, aromatic amines, sulfur dioxide, ascorbic acid, enediols, reductones, organic acids, Fe^2+^ ions, and several inorganic compounds such as hydrazine, hydroxylammonium chloride, ammonium iron sulfate, iron sulfate, manganese sulfate, potassium nitrite, and sodium cyanide [32,33].

The F-C method for measuring TPC generally involves incubating the sample with the F-C reagent (for between 1 and 8 min), followed by the addition of a sodium carbonate solution (at concentrations ranging from 5 to 35% *w*/*v*). The mixture is incubated (for 5 min to 2 h), either at room temperature or under controlled heating conditions (with higher temperatures generally allowing shorter incubation time). Subsequently, the absorbance is measured using a spectrophotometer, typically at wavelengths between 630 and 765 nm, with 765 nm the most commonly used (Table S1).

Various methodologies have been described for the quantification of TPC using the F-C assay. In general, GAL is the most commonly used standard, with results expressed as gallic acid equivalents (GAE), whereas CAFF and quercetin (QUER) are used less frequently, with results expressed as caffeic acid equivalents (CAE) and quercetin equivalents (QE) (Table S1). The concentrations and dilution factors employed are selected according to the expected phenolic content of the sample to ensure that the measured values fall within the calibration curve range.

#### 2.1.2. Chromatographic Techniques for the Identification and Quantification of Individual Phenolic Compounds

Alternatively, to complement the F-C assay for TPC determination, chromatographic techniques, specifically HPLC, can be used to quantify individual phenolic compounds, thereby enabling their identification and quantification. HPLC is the workhorse technique for phenolic separation and is widely preferred for both separation and quantification. Several factors influence HPLC analysis of phenolics, including sample purification, mobile phase composition, column, and detector type (Table 1).

In general, purified phenolic compounds are analyzed using an HPLC system equipped with a reversed-phase C18 column (RP-C18), a DAD or UV-Vis detector and acidified polar organic solvents in the mobile phase [34,35]. Acetonitrile and methanol, or mixtures of these with water, are the dominant mobile phases used in the HPLC quantification of phenolic compounds. Additionally, it is recommended to maintain the mobile phase pH within the range of 2 to 4 to prevent ionization of phenolics during the analysis. Therefore, acidified aqueous mobile phases predominantly contain formic acid, acetic acid, orthophosphoric acid, or trichloroacetic acid (Table 1).

The quantification and detection of phenolic compounds in OP extracts are generally performed using a C18 column. A clear relationship can be observed between the chromatographic system employed and both the column dimensions and particle size.

Conventional HPLC methods, particularly those coupled to UV, UV-Vis or DAD detectors, predominantly use C18 columns with lengths of 150–250 mm, internal diameters of 4.0–4.6 mm, and particle sizes of 5 μm. These configurations are associated with relatively high flow rates (typically 1 mL min^−1^) and longer analysis times, reflecting the lower efficiency of larger particles and the need for increased column length to achieve adequate resolution. In contrast, methods coupled to mass spectrometry (MS, TOF-MS, QTOF-MS, or MS/MS) tend to employ narrower columns (2.1–3.0 mm i.d.) and smaller particle sizes, typically between 1.7 and 3.0 μm. The reduction in particle size increases chromatographic efficiency and peak capacity, while the smaller internal diameter reduces solvent consumption and improves compatibility with electrospray ionization, leading to lower flow rates (0.2–0.5 mL min^−1^). The most advanced systems, particularly UHPLC-DAD, use sub-2 μm particles (1.7 μm) in short columns (100 × 2.1 mm), allowing a substantial reduction in analysis time while maintaining or improving separation performance. Overall, the data indicate a clear trend from long conventional columns packed with 5 μm particles in HPLC methods towards shorter and narrower columns packed with smaller particles in UHPLC and MS-based platforms, enabling faster analyses, higher separation efficiency, and improved detection sensitivity. For detection and quantification, UV-Vis and DAD are most frequently employed, typically operating within a wavelength range of 190 to 390 nm [35]. In general, HYT, TYR, and OLE are frequently quantified at 280 nm (Table 1). These compounds constitute the major phenolic constituents present in OP.

Most HPLC methods used for the analysis of phenolic compounds in OP extracts are performed at ambient column temperature. The highest temperature reported for HPLC analyses of OP extracts is 35 °C using columns packed with 5 µm particles [24,36]. For UHPLC, the maximum documented column temperature is 40 °C [30]. Although UHPLC systems commonly operate at higher temperatures and employ columns packed with smaller particles (≤2.5 µm) than HPLC, temperatures above 40 °C have not been reported for OP extracts. This limitation may be related to the thermal sensitivity of phenolic compounds, as higher temperatures can increase the risk of degradation. Column temperature plays an important role in chromatographic performance, since increasing temperature reduces mobile phase viscosity, enhances mass transfer, and shortens analysis time. In addition to column temperature, analysis time is another important parameter that influences chromatographic separation. For HPLC analysis, run times typically range from 18 to 75 min when a broader range of compounds is evaluated. In the case of UHPLC, an analysis time of 30 min has been reported (Table 1). These findings are consistent with Khoddami et al. [35], who reported that phenolic compound analysis times may vary between 10 and 150 min.

HPLC coupled with UV–Vis or DAD is a technique that enables rapid analysis of bioactive compounds. However, it has limitations, as it provides only limited structural information, such as UV-Vis spectra and tentative characterization of eluted peaks by comparing them with reference standards. Mass spectral fragmentation patterns offer valuable information, including fragmentation pathways that allow the identification of more complex constituents in the sample. Therefore, coupling chromatographic methods with mass spectrometry (MS) detection systems enables the rapid identification and quantification of unknown compounds, as MS is highly sensitive and provides high specificity through mass-selective detection. Among the available interfaces, electrospray ionization (ESI) is the most widely used in HPLC-MS system. ESI can operate in positive-ion mode (+ve), where protonation occurs, or negative-ion mode (−ve), where analytes are ionized through deprotonation, allowing more accurate compound characterization. MS involves three fundamental steps: ionization, mass analysis, and detection. Upon introduction of the analyte into the mass spectrometer, ionization occurs and the molecular mass of the target compounds is determined based on their mass-to-charge ratio (*m*/*z*) [35,37].

For the analysis of OP extracts, quadrupole (Q), triple quadrupole (TQ), ion trap (IT), time-of-flight (TOF), and quadrupole time-of-flight (QTOF) mass analyzers have been employed (Table 1). These systems separate ions according to their *m*/*z* and provide complementary capabilities for compound identification, quantification, and structural characterization. While Q and TQ analyzers are commonly used for targeted analysis and quantification, TOF and QTOF systems offer high mass accuracy and are particularly useful for compound identification and structural elucidation [38,39].

**Table 1:** Measurement Methodologies for Phenolic Compounds by Chromatography in Olive Pomace Extracts.

Compounds	Analytical Technique	Column, Flow Rate, T^a^	Mobile Phase	Gradient	Ref
HYT, TYR, OLE, CAFF, API, VAN and others	HPLC-UV 280 nm	C18 (250 × 4.6 mm; 5 μm), 1 mL/min, 30 °C	A: Water with 1% acetic acid B: Acetonitrile: Methanol (1:1 *v*/*v*)	O min 5% B, 0–25 min 30% B, 25–35 min 40% B, 35–40 min 48% B, 40–50 min 70% B, 50–55 min 100% B, 55–60 min 100% B, 60–70 min 5% B, 70–72 5% B.	[25]
HYT, OLE, CAFF, LUT and others	HPLC-UV-Vis 280 nm	C18 (150 × 4.6 mm; 5 μm), 1 mL/min	A: Water with 0.01% acetic acid B: Methanol: Acetonitrile: Acetic acid (95:5:1 *v*/*v*/*v*)	0–2 min 5% B, 2–8 min 25% B, 8–10 min 40% B, 10–20 min to 50% B; 20–30 100% B; 30–52 min 100% B; 52–70 min 5% B.	[40]
HYT, TYR, OLE, CAFF, API, VAN, FER, LUT and Others	HPLC-DAD 230, 280, 325, and 350 nm	C18 (250 × 4 mm; 5 μm), 1 mL/min	A: Water with 0.2% phosphoric acid B: Acetonitrile: Methanol (1:1 *v*/*v*)	0–40 min 50% B; 40–45 min 60% B; 45–55 min 100% B; 55–57 min 100% B.	[41]
HYT, TYR, OLE, VAN and others	HPLC-DAD 254, 280 and 340 nm	C18 (250 × 4.6 mm; 5 μm)	A: Water with 0.01% trichloroacetic acid B: Acetonitrile	0 min 5% B, 0–30 min 25% B, 30–45 min 50% B, 45–47 min 100% B, 50 min 25% B, 50–52 min 5% B. 52–55 min 5% B.	[42,43]
HYT	HPLC-UV-Vis 280 nm	C18 (250 × 4.6 mm; 5 μm), 1 mL/min	A: Water with 5% acetic acid B: Methanol	0 min 5% B, 0–3 min 10% B, 3–18 min 25% B, 18–19 min 29% B, 19–24 min 30% B, 24–30 min 31% B, 30–31 min 35% B, 31–41 min 45% B, 41–51 min 55% B, 51–61 min 65% B, 61–67 min 100% B, 67–70 min and 5% B.	[44]
HYT, TYR, OLE, and Others	HPLC-UV-Vis-TQ-MS/MS ESI (-) 280 nm	C18 (150 × 2.1 mm; 5 μm), 0.2 mL/min	A: Water: acetonitrile (90:10 *v*/*v*) with 0.1% formic acid B: Acetonitrile with 0.1% formic acid	0 min 0% B, 0–3 min 5% B, 3–18 min 20% B, 18–19 min 24% B, 19–24 min 25% B, 24–30 min 26% B, 30–31 min 30% B, 31–61 min 60% B, 61–67 min 100% B.	[44]
HYT, TYR, OLE, and Others	HPLC-DAD-IT-MS^n^ ESI (-) 280 nm	C18 (150 × 2.1 mm; 5 μm), 0.2 mL/min	A: Water: acetonitrile (90:10 *v*/*v*) with 0.1% formic acid B: Acetonitrile with 0.1% formic acid	0 min 0% B, 0–3 min 0% B, 3–10 min 10% B, 10–30 min 20% B, 30–35 min 25% B, 35–50 min 50% B, 50–60 min 0% B.	[44]
HYT, OLE (aglycone), API, LUT and others	HPLC-DAD-TOF-MS ESI (-) 240 and 280 nm	C18 (150 × 4.6 mm; 1.8 μm), 0.8 mL/min, 25 °C	A: Water with 0.25% acetic acid B: Methanol	0 min 5% B, 0–7 min 35% B, 7–12 min 45% B, 12–17 min 50% B, 17–22 min 60% B, 22–25 min 95% B, 25–27 min 5% B, 27–32 min 5% B.	[45]
HYT and TYR	HPLC-UV-Vis 280 nm	C18 (30 × 4.6 mm; 3.5 μm), 1 mL/min	A: Water: Methanol: Acetonitrile, (70:24:6 *v*/*v*/*v*); pH 7.0	Isocratic.	[46]
HYT, OLE, CAFF, RUT, LUT and VA	HPLC-DAD 280 and 330 nm	C18 (250 × 4.6 mm; 5 μm)	A: Water: acetic acid (94:6 *v*/*v*) with 2 mM sodium acetate B: Acetonitrile	0 min 0% B, 0–25 min 50% B (0.8 mL/min), 25–27 min 100% B (0.8 mL/min–1.2 mL/min), 27–40 min 100% B (1.2 mL/min), 40–41 min 100–0% B (1.2 mL/min–0.8 mL/min), 41–45 min 0% B (0.8 mL/min)	[20]
HYT, TYR, OLE, GAL, CAFF, API, RUT, FER, LUT, QUER, and others	HPLC-DAD 254, 280, 320 and 370 nm	C18 (150 × 4.6 mm; 5 μm), 1 mL/min, 30 °C	A: Water with 0.1% formic acid B: Acetonitrile	0–2.7 min 5% B, 2.7–10.7 min 30% B, 10.7–11 min 32% B, 11–15 min 40% B, 15–15.5 min 50% B, 15.5–16 min 50% B, 16–16.5 min 30% B, 16.5–17 min 15% B, 17–17.5 min 5% B, 17.5–18 min 5% B.	[47]
HYT and OLE	HPLC-UV 280 nm	C18 (250 × 4.6 mm; 5 um), 1 mL/min, 25 °C	A: Water with 0.5% glacial acetic acid B: Acetonitrile	O min 5% B, 0–30 min 20% B, 30–40 min 30% B, 40–45 min 35% B.	[48]
HYT, TYR and pinoresinol	HPLC-DAD/FLD DAD 280, 320 and 335 nm/FLD 280 ex. and 330 em.	C18 (25 × 4.6 mm; 5 μm), 1 mL/min, 20 °C	A: Water with 1% acetic acid B: Methanol	0 min 10% B, 0–20 min 30% B, 20–50 min 75% B, 50–63 min 100% B, 63–65 min 10% B, 65–70 min 10% B.	[49]
HYT, TYR, OLE and others	HPLC-DAD-IT-MS^n^ ESI (-) 280, 320 and 335 nm	C18 (25 × 4.6 mm; 5 μm), 0.5 mL/min	A: Water with 1% acetic acid B: Methanol	0 min 10% B, 0–20 min 30% B, 20–50 min 75% B, 50–63 min 100% B, 63–65 min 10% B, 63–70 min 10% B.	[49]
HYT, TYR, OLE, CAFF, API, RUT, VAN, LUT, VERB and others	HPLC-DAD 280 nm	C18 (250 × 4.6 mm; 5 um), 1 mL/min, 35 °C	A: Water pH 2.5 adjusted with 0.15% phosphoric acid B: Methanol	0 min 10% B, 0–10 min 30% B, 10–20 min 30% B, 20–30 min 40% B 30–35 min 40% B, 35–40 min 50% B, 50–55 min 60% B, 55–60 min 70% B, 60–65 min 100% B, 65–75 min 10% B.	[36]
HYT, TYR, OLE, CAFF, API, VAN, LUT and others	HPLC-DAD-TOF-MS ESI (-) 240–280 nm	C18 (150 × 4.6 mm; 1.8 μm), 0.5 mL/min, 25 °C	A: Water with 0.5% acetic acid B: Methanol	0 min 5% B, 0–7 min 35% B, 7–13 min 45% B, 13–18.5 min 50% B, 18.5–22 min 60% B, 22–29 min 95% B, 29–36 min 5% B.	[22]
HYT, maslinic acid and oleanolic acid	HPLC-UV-Vis 210 nm	C18 (250 × 4.6 mm; 5 μm), 1 mL/min, 30 °C	A: Water with 1% Acetic acid B: Methanol	Isocratic 9% A and 91% B.	[27]
HYT, TYR, VAN, LUT, VERB, VA and others	HPLC-DAD 254, 280 and 340 nm	C18 (250 × 4.6 mm; 5 um), 1 mL/min, 25 °C	A: Water pH 2.5 adjusted with 0.01% trifluoroacetic acid B: Acetonitrile	0 min 5% B, 0–30 min 25% B, 30–45 min 50% B, 45–47 min 100% B, 47–52 min 5% B, 52–55 min 5% B.	[50]
HYT	HPLC-DAD 280 nm	C18 (250 × 4.6 mm; 5 μm), 1 mL/min	A: Water with 0.2% orthophosphoric acid B: Methanol C: Acetonitrile	0 min 96% A, 2% B, 2% C, 0–40 min 25% B and 25% C, 40–45 min 30% B and 30% C,45–60 min 50% B and 50% C, isocratic at 50% for 8 min, and then 50 to 2% in 4 min.	[51,52]
HYT, TYR, OLE, LUT and others	HPLC-IT-MS/MS ESI (-)	C18 (50 × 2.1 mm; 2.7 μm), 0.35 mL/min	A: Water with 0.1% formic acid B: Acetonitrile with 0.1% formic acid	0 min 4% B, 0–1 min 7% B, 1–15 min 30% B, 15–19.5 min 40% B, 19.5–24 min 100% B, isocratic at 100% for 2 min, 100 to 4% in 1.5 min, and isocratic at 4% for 7 min.	[51,52]
HYT OLE, and TYR	HPLC-DAD 280 nm	C18 (250 × 4.6 mm; 5 μm), 1 mL/min, 35 °C	A: Water pH 3 adjusted with phosphoric acid B: Methanol	0 min 10% B, 0–10 min 20% B, 10−16 min 20% B, 16−20 min 30% B, 20−25 min 30% A, 25−35 min 40% B, 35−40 min 40% B, 40−45 min 45% B, 45−55 min 45% B, 55−60 min 60% B, 60−65 min 70% B, 65−70 min 100% B.	[24]
HYT, TYR, OLE and others	HPLC-DAD-TQ-MS/MS ESI (-) 210–600 nm	C18 (250 × 4 mm; 5 μm), 0.3 mL/min, 35 °C	A: Water with 0.5% formic acid B: Methanol	0–15 min 30% B, 15–45 min 40%, 45–60 min 40% B, 60–75 min 45% B, 75–105 min 45% B, 105–125 min 30% B.	[24]
HYT, OLE and Others	HPLC-TOF-MS ESI (-)	C18 (150 × 4.6 mm; 1.8 µm), 0.5 mL/min, 25 °C	A: Water with 0.25% acetic acid B: Methanol	0 min 5% B, 0–7 min 35% B, 7–12 min 45% B, 12–17 min 50% B, 17–22 min 60% B, 22–25 min 95% B, 22–27 min, 5% B.	[53]
HYT, TRY, OLE, CAFF, API, RUT, VAN, FER, LUT and others	HPLC-DAD 280, 320 and 365 nm	C18 (250 mm × 4.6; 5 μm), 1 mL/min	A: Water with 3% acetic acid B: Methanol: acetonitrile (1:1 *v*/*v*)	0 min 5% B, 0–25 min 30% B, 25–35 min 35% B, 35–40 min 40% B, 40–50 min 70% B, 50–55 min 100% B, 55–60 min 100% B, 60–65 min 5% B, 65–70 min 5% B.	[54]
TYR, OLE, RUT, LUT, VERB and Others	HPLC-DAD 210, 240, 280 and 350 nm	C18 (150 × 3.0 mm; 2.7 μm), 0.4 mL/min, 26 °C	A: Water pH 3.2 adjusted with acid formic B: Acetonitrile	0.1 min 5% B, 0.1–40 min 40% B, 40–45 min 40% B, 45–50 min 70% B, 50–60 min 70% B, 60–65 min 100% B, 65–68 min 100% B, 68–70 min 5% B.	[55]
HYT and LUT	HPLC-UV 254 and 280 nm	C18 (100 × 3.0 mm; 2.6 μm), 0.4 mL/min, 30 °C	A: Water with 0.1% formic acid B: Acetonitrile	0 min 5% B, 0–4 min 7% B, 4–10 min 50% B, 10–11 min 80% B, 11–15 min 80% B, 15–16 min 10% B, 16–18 min 10% B.	[56]
HYT, TYR, OLE, CAFF, API, RUT, VAN, FER, QUER and others	UHPLC-DAD n/s	C18 (100 mm × 2.1; 1.7 μm), 0.4 mL/min, 40 °C	A: Water with 0.1% formic acid B: Acetonitrile	0–5.5 min 5% B, 5.5–17 min 60% B, 17−18.5 min 100% B, 18.5–30.0 min 5% B.	[30]
HYT, VERB and others	HPLC-DAD 280 and 240 nm	C18 (250 × 4.6 mm; 5 μm), 1 mL/min	A: Water pH 3 adjusted with phosphoric acid B: Methanol	0 min 10% B, 0–10 min 30% B, 10–20 min 30% B, 20–30 min 40% B, 30–35 min 40% B, 35–40 min 50% B, 40–45 min 60% B, 45–50 min 70% B, 50–55 min 100% B, 55–70 min 10% B.	[57]
HYT, TYR, OLE, CAFF, API, LUT and others	HPLC-DAD/FLD 280, 240 and 350 nm	C18 (250 × 4.6 mm; 5 µm), 1 mL/min	A: Water with 0.5% acetic acid B: Methanol: acetonitrile (1:1 *v*/*v*)	0 min 5 % B, 30 min 25 % B, 50 min 75 % B, 55 min 100 % B, 60 min 100 % B, 63 min 5 % B.	[58]
HYT, TYR, OLE, GAL, CAFF, VAN and others	HPLC-DAD-Q-MS-ESI (-) 280 nm	C18 (150 × 4.6 mm; 3 µm), 1 mL/min	A: Water with 0.1% formic acid B: Acetonitrile with 0.1% formic acid	0 min 5% B, 15 min 10% B, 25 min 20% B, 40 min 50% B, 41 min 100% B, 48 min 100% B, 49 min 5% B, 56 min 5% B.	[59]
HYT, OLE, API, LUT, VERB and others	HPLC-QTOF-MS- ESI (-)	C18 (150 × 4.6 mm; 1.8 µm), 0.5 mL/min	A: Water with 0.25% acetic acid B: Methanol	0 min 5% B, 7 min 35% B, 13 min 45% B, 18.5 min 50% B, 22 min 60% B, 29 min 95% B, 36 min 5% B.	[60]
HYT, OLE, LUT, VERB and others	HPLC-QTOF-MS/MS-ESI (-)	C18 (100 × 2.1 mm; 1.8 µm), 0.5 mL/min, 40 °C	A: Water with 0.1% formic acid B: Acetonitrile with 0.1% formic acid	0–7 min 30% B, 9 min 80% B, 11 min 100% B, 13 min 100% B, 14 min 0% B, 17 min 0% B.	[61]

n/s: Not specified. Note: When not explicitly reported, analysis temperature was assumed to be room temperature. HPLC: high-performance liquid chromatography; DAD: diode array detector; FLD: fluorescence detector; UV-Vis: Ultraviolet-visible detector; TQ: Triple Quadrupole; IT: Ion Trap; TOF: Time-of-Flight; Q: Quadrupole; QTOF: Quadrupole Time-of-Flight; MS: mass spectrometry; ESI: electrospray ionization. HYT: hydroxytyrosol; TYR: Tyrosol; OLE: Oleuropein; GAL: gallic acid; CAFF: caffeic acid; API: apigenin; RUT: Rutin; VAN: Vanillic acid; FER: Ferulic acid; LUT: Luteolin; QUER: Quercetin; VERB: verbascoside; VA: vanillin.

Metrological information reported in OP extraction studies was scarce, as only two studies provided analytical validation parameters. Nevertheless, the reported results indicated satisfactory chromatographic performance, with good precision and accuracy and limits of detection and quantification in the sub-μg g−1 (*w*/*w*) range. A summary of these validation parameters is presented in Supplementary Table S2.

#### 2.1.3. Spectrophotometric Assays for the Assessment of Antioxidant Activity

OP extracts are primarily rich in phenolic compounds, which are largely responsible for their antioxidant activity. Antioxidant activity has been assessed using several widely applied methods, including ORAC, DPPH, FRAP, ABTS and CUPRAC. These assays have been employed in numerous studies (Table S3) to evaluate the antioxidant capacity of the bioactive compounds present in OP extracts.

- 2,2-Diphenyl-1-picrylhydrazyl (DPPH) Assay.

DPPH scavenging assay is the oldest indirect method for determining antioxidant activity (1950s). DPPH is a stable organic nitrogen radical containing an unpaired electron and has an intense purple color. It is used to quantify and determine the antioxidant potential of both individual phenolics and food or biologically relevant samples. The reaction results in a noticeable discoloration from purple to yellow, due to the reduction of the stable radical DPPH to diphenyl-picrylhydrazine, leading to a reduction in the absorbance of the reaction mixture measured at 515–520 nm. This decrease in absorbance, accompanied by the color change, correlates with the hydrogen-donating capacity, concentration, and activity of the antioxidant present in the sample (Table S3). The antioxidant activity of the sample is measured until the absorbance remains constant [62,63,64]. The results of antioxidant activity assays are typically expressed as mg Trolox equivalents (TRE) per gram (or mL), or as a half-inhibitory concentration (IC50) value (µg/mL), which corresponds to the extract concentration required to reduce the initial DPPH concentration by 50%; or as a percentage of inhibition. Trolox, a Hoffman-La Roche trade name for 6-hydroxy-2,5,7,8-tetramethylchroman-2-carboxylic acid, is a water-soluble analogue of vitamin E commonly used as a reference antioxidant compound [64]. The specific formula used to calculate % inhibition varies among studies, but it generally involves comparing the absorbance of the sample with that of the DPPH control solution and a blank or reference solution.

- Ferric Reducing Antioxidant Power (FRAP) assay.

FRAP is a colorimetric method used to evaluate the antioxidant capacity of compounds. The assay measures the ability of antioxidants to reduce the ferric ion in the Fe3+-TPTZ (ferric 2,4,6-tripyridyl-s-triazine) complex. When reacting with an antioxidant compound, a single electron is transferred to the ferric complex [Fe^3+^-(TPTZ)2]^3+^, forming the intensely blue-colored ferrous complex [Fe^2+^-(TPTZ)2]^2+^ in an acidic medium. This transformation from a colorless to a blue solution occurs under acidic conditions, typically at pH 3.6, which maintains iron solubility. The maximum absorbance intensity is observed at 593 nm. The FRAP reagent is prepared by mixing acetate buffer (0.3 M), TPTZ solution (10 mM in HCl), and ferric chloride (20 mM) in a 10:1:1 ratio (Table S3). Lower pH levels reduce the ionization potential, which facilitates electron transfer, while simultaneously increasing the redox potential. This shift alters the primary reaction mechanism, enhancing the sensitivity of the assay under acidic conditions [62,65]. FRAP assay results are commonly expressed as µmol ferrous sulfate equivalents (FSE) per gram (or µmol ferrous ion equivalents (Fe(II)/g)), or mg Trolox equivalents (TRE) per gram. These units reflect the antioxidant capacity of the sample based on its ability to reduce ferric ions under the assay conditions.

- Oxygen Radical Absorbance Capacity (ORAC) assay.

The ORAC assay evaluates the ability of antioxidants to inhibit the propagation of radical chain reactions by monitoring the suppression of peroxyl radical oxidation. Due to its relevance to biological systems, ORAC values are considered by many researchers as a reliable benchmark for antioxidant efficiency. Common peroxyl radicals are thermally generated (at 37 °C) in the presence of oxygen using azo compounds such as lipophilic α,α-azobis(isobutyronitrile) (AIBN), 2,2′-azobis(2-amidinopropane) hydrochloride (ABAP) and 2,2′-azobis(2,4-dimethylvaleronitrile) (AMVN). However, the most frequently used compound is the hydrophilic 2,2′-azobis(2-amidinopropane) dihydrochloride (AAPH). These radicals interact with a fluorescent probe, typically fluorescein, leading to fluorescence decay [31,65]. In the presence of chain-breaking antioxidants, the decay of fluorescence is inhibited, reflecting the antioxidant’s capacity to neutralize peroxyl radicals and interrupt the oxidative chain reaction [62]. The assay is typically performed using commercially available 96-well polypropylene fluorescence plates, and the decrease in fluorescence is monitored at excitation/emission wavelengths of 485/520 nm [63].

For hydroxyl radical evaluation, systems such as H_2_O_2_–CuSO_4_ are used, with phycoerythrin acting as a redox-sensitive fluorescent indicator [31]. Results are typically expressed as TRE per gram of dry weight (DW) [21,24].

- 2,2′-Azinobis (3-Ethylbenzothiazoline-6-Sulfonic Acid) (ABTS) assay.

The ABTS assay evaluates antioxidant capacity by measuring the ability of compounds to neutralize the stable radical cation ABTS^+^, a blue-green chromophore with maximum absorbance at 734 nm. The radical is generated by oxidizing ABTS with potassium persulfate. Antioxidants reduce ABTS^+^ through hydrogen donation, leading to a measurable decrease in absorbance [31,63,66]. ABTS^+^ is typically generated by reacting 7 mM ABTS with 2.45 mM potassium persulfate, followed by incubation and dilution with methanol to prepare the working solution. The sample or standard (e.g., ascorbic acid or Trolox) is mixed in a 1:1 ratio with the ABTS reagent, incubated at room temperature for 6 min, and absorbance is measured at 734 nm (Table S3) [63]. The degree of discoloration depends on the reaction time, antioxidant concentration, and intrinsic activity of the sample. Although antioxidant capacity is typically expressed as TRE per gram or milliliter (mg TRE/g or mg TRE/mL), several studies also report results as a percentage of inhibition [54]. These alternative expressions depend on the methodology and are generally based on the comparison between the absorbance of the sample and that of a control or blank solution.

- Cupric Ion Reducing Antioxidant Capacity (CUPRAC) assay.

The CUPRAC assay is based on the reduction of cupric ions (Cu^2+^) to cuprous ions (Cu^+^). A ligand is used to form a copper–ligand complex, which facilitates absorbance measurement. Neocuproine (2,9-dimethyl-1,10-phenanthroline) is the ligand most commonly employed in this assay. The Cu^2+^–neocuproine complex acts as a chromogenic agent and exhibits a light blue color. Upon interaction with a reducing agent, this complex is converted into a Cu^+^–neocuproine complex, which displays a characteristic orange-yellow color with a maximum absorbance at 450 nm [31].

#### 2.1.4. Comparative Assessment of Analytical Methodologies

Both spectrophotometric and chromatographic methodologies have been widely employed for the characterization of OP extracts. While spectrophotometric assays provide rapid and cost-effective screening of phenolic content and antioxidant activity, chromatographic techniques offer a more detailed characterization of individual phenolic compounds.

Methods such as the F–C assay, DPPH, ABTS, FRAP, ORAC, and CUPRAC provide valuable information regarding total phenolic content and antioxidant activity and are commonly employed as screening tools. However, these methodologies are generally based on non-specific chemical reactions and may be affected by matrix effects and interfering compounds present in the extracts. Consequently, the obtained values may overestimate or underestimate the actual phenolic content or antioxidant capacity depending on the extraction conditions and sample composition. Factors such as extract composition, color, turbidity, and the presence of interfering compounds may affect spectrophotometric measurements, particularly in assays based on radical discoloration, including DPPH and ABTS [31]. The magnitude of these interferences may vary according to the extraction medium employed, since aqueous, hydroalcoholic, and DES-based extracts can differ considerably in viscosity, color, turbidity, and the presence of co-extracted compounds. The main advantages and limitations of these methodologies are summarized in Table S4.

Historically, the Folin–Ciocalteu assay has been the most widely employed method for estimating total phenolic content due to its simplicity, rapid execution, and low analytical cost. Although the development of modern chromatographic techniques has enabled the identification and quantification of individual phenolic compounds with much greater specificity, TPC measurements continue to be extensively reported in extraction studies. This persistence is largely attributable to the ability of the F–C assay to provide a rapid estimation of the reducing capacity associated with phenolic-rich extracts, facilitating comparisons among extraction conditions and studies. Therefore, rather than being replaced by chromatographic methodologies, the F–C assay remains a complementary tool whose value lies in screening applications and comparative purposes, whereas chromatographic techniques provide the detailed compositional information required for a comprehensive characterization of phenolic extracts.

To minimize potential interferences, extraction protocols frequently include pretreatment and clarification steps such as defatting, centrifugation, and filtration prior to analysis. Nevertheless, spectrophotometric assays do not provide information on the identity or concentration of individual phenolic compounds. For this reason, their results are often complemented by chromatographic techniques, mainly HPLC and UHPLC coupled to UV, DAD, or mass spectrometric detectors. These methods enable the separation, identification, and quantification of specific phenolics, providing a more accurate assessment of extract composition and facilitating the evaluation of how extraction conditions affect the recovery of individual bioactive compounds.

As shown in Figure S1, both analytical approaches have been extensively employed throughout the last decade, with chromatographic methods becoming increasingly prevalent since 2018. Nevertheless, spectrophotometric assays continue to be frequently reported due to their practicality as rapid and economical screening tools. Therefore, the combined use of spectrophotometric and chromatographic methodologies provides a more comprehensive characterization of OP extracts and a more robust evaluation of extraction performance. Likewise, the combined use of complementary antioxidant assays is often recommended, as different methodologies rely on distinct reaction mechanisms and may provide different estimations of antioxidant capacity for the same sample.

### 2.2. Phenolic Compound Extraction from Olive Pomace: Technologies and Extraction Efficiency Assessment

The valorization of OP through phenolic compound recovery involves several stages, including pretreatment, extraction, and analytical evaluation of extraction efficiency. As summarized in Figure 2, both conventional and green extraction technologies have been investigated to maximize the recovery of bioactive compounds while improving process sustainability. The following sections describe the main pretreatment strategies, extraction approaches, and the parameters commonly used to assess extraction performance (Table 2).

#### 2.2.1. Olive Pomace Pretreatments

Several studies have underscored the importance of applying pretreatment steps prior to the extraction of phenolic compounds. As previously mentioned, pretreatment strategies may include sample dehydration, along with milling and homogenization to ensure sample representativeness and uniformity. The removal of residual lipid material is also commonly employed to enhance the extraction efficiency of hydrophilic compounds. In addition, some studies report on the use of hydrothermal pretreatments aimed at improving the extractability and overall recovery of phenolic constituents.

Drying treatments are widely employed due to the high moisture content of OP (60–80%), which increases its susceptibility to microbial activity. However, drying may adversely affect the stability of valuable bioactive compounds, such as phenols and tocopherols, by promoting free radical formation, oxidative reactions, and the hydrolysis of thermolabile molecules. Therefore, careful control of both drying temperature and exposure time is essential to minimize the loss of these compounds [21,79,80,81,82]. For this reason, hot air drying, typically conducted at temperatures up to 60 °C (between 35 and 60 °C), is the most commonly used technique for OP (Table 2).

Drying temperature is a critical factor affecting the stability of phenolic compounds and therefore warrants specific evaluation. Studies focusing exclusively on OP drying have shown that, in destoned OP, hot-air drying at 80 °C for 130 min results in a compound-dependent response. Under these conditions, significant reductions were observed in VAN, CAFF, VERB, luteolin-7-glucoside, RUT, and LUT, as well as in antioxidant capacity, compared to fresh OP. Notably, significant losses were also detected using other drying techniques, such as freeze-drying carried out for 69 h, indicating that temperature, processing time, and sample handling are critical variables affecting phenolic compound degradation [83]. Overall, freeze-drying for approximately 48 h has been recognized as an effective approach for preserving nutritional and phenolic compounds and is therefore widely employed at the laboratory scale. However, its long processing time and high operating costs limit its industrial application [82].

Defatting step was considered essential, as lipids act as interfering agents in the extraction of hydroalcoholic compounds. This procedure is commonly performed prior to the extraction of phenolic compounds, since non-polar constituents such as fatty acids, triacylglycerols, fat-soluble vitamins, and pigments can interfere with both the extraction efficiency and the accurate quantification of phenolics [84]. Commonly used solvents for lipid removal include n-hexane. In general, the solvent-to-sample ratio is not consistently reported; however, studies that specify this parameter indicate ratios of 1:5, *w*/*v* or 1:0.5, *v*/*v* (Table 2). Additionally, some studies report oil removal using petroleum ether, although no information regarding the solvent-to-sample ratio is provided [46,75]. These solvents effectively eliminate lipophilic components without significantly altering the phenolic profile. Another reported technique is fat extraction using supercritical carbon dioxide (SC-CO_2_), an environmentally friendly method that avoids the use of organic solvents. The proposed procedure involves extraction with CO_2_ at a flow rate of 10.5 kg per hour for 3 h, at 20 °C and 6 MPa [24].

Another pretreatment technique is hydrothermal treatment, which facilitates the extraction of valuable compounds and promotes liquid/solid separation into two distinct phases (solid and liquid). OP has been treated with techniques aimed at oil extraction and improving the recovery of bioactive compounds in the aqueous phase. Hydrothermal treatments (steam explosion [SE] and subcritical water [SCW]) were applied prior to aqueous-phase separation by centrifugation. SE is typically performed under temperatures ranging from 160 to 260 °C and pressures between 0.69 and 4.83 MPa. In contrast, SCW extraction employs liquid water at temperatures between 100 and 374 °C while maintaining sufficient pressure to preserve the liquid state. These techniques resulted in a 1% increase in TPC in pretreated OP extracts compared with untreated samples, demonstrating the effectiveness of hydrothermal pretreatment in improving phenolic compound extraction [85].

Hydrothermal treatment promotes the disruption of the lignocellulosic matrix through the hydrolysis of structural polysaccharides and modifications of lignin, thereby increasing cell wall permeability and facilitating the release of bound phenolic compounds. In addition to enhancing phenolic extractability, hydrothermal pretreatments may significantly modify the composition of individual phenolic compounds. Previous studies have shown that treatment severity, particularly temperature and residence time, promotes the hydrolysis of complex secoiridoids and the formation of simpler phenolics. For example, increased concentrations of hydroxytyrosol, tyrosol, and 3,4-dihydroxyphenylglycol have been associated with the breakdown and solubilization of more complex phenolic structures. Conversely, oleuropein concentrations decrease during treatment due to degradation reactions, while compounds such as demethyloleuropein and demethyl ligstroside may be formed as intermediate transformation products. Higher treatment severity may also promote the degradation of certain phenolic compounds and the formation of secondary products, such as hydroxymethylfurfural, derived from hexose degradation. Oleuropein and ligustroside, the main bitter secoiridoid glycosides present in OP, are particularly susceptible to hydrolytic and thermal transformations during hydrothermal pretreatment, resulting in the formation of simpler phenolic compounds, including hydroxytyrosol- and tyrosol-related derivatives. Therefore, hydrothermal pretreatment acts through a dual mechanism: (i) disruption of the lignocellulosic matrix, which enhances mass transfer and phenolic release, and (ii) hydrolytic and thermal transformation of complex phenolic structures, which modifies the phenolic profile and may increase the concentration of certain bioactive phenolics [42,86,87].

Finally, other sample pretreatments include milling, sieving, and centrifugation to remove residual oil or water. Additionally, the removal of the olive stone (or pit) and/or skin is sometimes performed to improve extraction efficiency and reduce matrix interference (Table 2).

#### 2.2.2. Phenolic Compound Extraction as a Valorization Strategy

Various extraction techniques have been employed for the extraction of phenolic compounds from OP, including conventional methods such as SLE using organic solvent, water or aqueous–organic solvent mixtures, as well as LLE. In addition, several green extraction techniques, such as UAE, MAE, SFE, PLE, HHPAE, OHE and PEF, have been explored, together with the use of DES in both conventional extraction methods and emerging extraction technologies (Table 2). These methods differ in terms of extraction efficiency, scalability, and environmental impact, and are selected according to the desired yield, compound stability, and intended application.

Conventional methods for extracting phenolic compounds generally require longer processing times and higher energy consumption. This has driven the development of eco-friendly alternatives aimed at improving extraction efficiency and sustainability [47]. Within this context, the use of environmentally friendly solvents and alternative extraction technologies has been increasingly investigated to improve the recovery of bioactive compounds while reducing environmental impacts. The main characteristics, advantages, drawbacks, and extraction efficiencies of these technologies are reviewed and compared in the following sections.

##### Conventional Solvent-Based Extraction Methods

SLE is one of the most widely used extraction methods due to its simplicity, effectiveness, and broad applicability. This technique involves the use of different solvents, such as methanol, ethanol, acetone, water or aqueous-organic solvent mixtures, and can be applied to either fresh or dried samples. Extraction is typically performed using an appropriate solvent in combination with conventional extractors or homogenizers. Among the most critical variables affecting extraction performance are temperature, extraction time, solvent type, the solid-to-solvent ratio, and agitation speed [88].

Agitation speed and temperature are key parameters influencing TPC extraction efficiency, primarily due to enhanced mass transfer. However, it is also essential to consider the raw material employed, as phenol concentration can vary significantly among different varieties, and according to harvest season, storage duration, and other related factors [20,24]. TPC values of 78.2 mg GAE/g DW were reported using water as the solvent for 40 min and room temperature [44], whereas substantially lower TPC values (12.24 mg GAE/g DW) were obtained using water at 170 rpm, 30 min and 83 °C [30]. Given the similarity of the operating conditions employed, these differences in TPC are likely more closely related to olive variety, harvest season, or sample drying pretreatments than to the extraction technique itself.

Gómez-Cruz et al. [67] identified the temperature as a statistically significant factor in the extraction of phenolic compounds. The authors applied an experimental design varying temperature (25–85 °C), extraction time (30–90 min), and solid concentration (5–25%, *w*/*v*). Under these conditions, the optimal parameters were determined to be 85 °C, 90 min and 10% (*w*/*v*) using water as the extraction solvent, resulting in a TPC of 44.5 mg GAE/g. Additionally, when comparing different temperatures under the same extraction time and solid concentration (90 min and 15% *w*/*v*), a TPC of 37 mg GAE/g was obtained at 85 °C, whereas at 25 °C the TPC reached only 29.7 mg GAE/g. These results further confirm the positive influence of temperature on the solubilization and release of phenolic compounds from the matrix.

In addition, longer extraction times have been associated with greater extraction of phenolic compounds [40,70]. In this sense, Lafka et al. [40] reported that an extraction duration of 3 h was optimal for maximizing phenolic recovery when using mechanical agitation and solvents such as ethanol, methanol, or water. Extending the extraction period beyond 3 h resulted in a decline in the antioxidant potential of the phenolic extracts, likely due to prolonged exposure to environmental factors that promote degradation. The authors also observed that extraction efficiency increased at pH 2.0, indicating enhanced solubility under strongly acidic conditions. This effect may be attributed to protein precipitation at low pH, which promotes the release of phenolic compounds otherwise bound to the cell wall matrix [70]. Under these conditions (pH 2.0, a solid-to-liquid ratio of 1:5 (*w*/*v*), and an extraction time of 3 h), ethanol-based extraction provides the highest antioxidant activity among the solvents evaluated, reaching 59.8% DPPH inhibition. This value was slightly higher than that obtained using methanol, which exhibited the second-highest antioxidant activity, reaching 58.7% DPPH inhibition.

These findings are consistent with those of other studies, which have identified ethanol and methanol as particularly suitable solvents for the extraction of phenolic compounds, achieving high TPC and strong antioxidant activity. This is attributed to the higher affinity and solubility of phenolic compounds in polar solvents due to the presence of hydroxyl groups in their structure. In contrast, a study comparing different solvents, including water, acidified water containing 0.5% acetic acid, 50% acetone, and ethanol solutions at 20% and 50%, under agitation found no significant differences in TPC between aqueous organic solvents and water (TPC with 50% ethanol: 39.5 mg GAE/g OP; TPC with water: 38.1 mg GAE/g OP). Since no statistically significant differences were observed, water was selected as the extraction solvent due to its eco-friendly, safe, and sustainable nature, suggesting that water may represent a potentially optimal solvent for solid–liquid extraction processes, depending on the conditions [67].

Several studies have highlighted the importance of olive cultivar variability, reporting significant differences in the concentration of bioactive compounds. A study conducted by Zagmutt et al. [46] reported a higher TPC in the Barnea variety (275 mg QE/g fresh weight (FW)) compared with Arbequina (156 mg QE/g FW) using SLE with 80% methanol. Additionally, the Picual variety has been reported to exhibit the highest total phenol content, along with elevated levels of GAL and RUT hydrate. In contrast, the Frantoio variety shows a higher concentration of HYT, highlighting the marked influence of genetic and varietal factors on the phenolic profile of olive-derived materials [89]. In other studies, higher phenol extraction yields in the Arbequina variety compared with Nevadillo were reported [70,84]. Moreover, phenol extraction rates are influenced not only by cultivar but also by harvest year, with varietal, seasonal, geographic, and agronomic factors shaping the phenolic profile of olives. Cultivar variability also affects oil content, as higher oil levels tend to reduce phenol extraction efficiency. This reduction is likely due to interference from non-polar compounds, such as fatty acids and triglycerides, during phenolic quantification, which can hinder accurate recovery of hydrophilic phenolics [84].

In addition to SLE, LLE has also been employed for the extraction of phenolic compounds from OP. Typically, LLE is performed after an initial SLE step or following centrifugation to separate the liquid phase from the OP. Soberón et al. [70] evaluated LLE following a previous SLE step carried out through four successive extractions (the first at a 1:2 solid-to-solvent ratio (*w*/*v*), and the subsequent three at a 1:1 ratio, *w*/*v*), each performed for 120 min at 4 °C under magnetic stirring at 1500 rpm. The recovered liquid fraction was subsequently subjected to LLE using ethyl acetate, reporting an optimal pH of 2.0. This condition may be attributed to protein precipitation and the release of cell-wall-bound phenolics, as well as to the increased solubility of phenolic compounds under acidic conditions. Under these conditions, LLE yielded TPC values ranging from 0.5 to 1.2 g CAE/g after 120 min at 20 °C.

Studies employing a preliminary hydrothermal treatment (160 °C; 60 min) followed by LLE with ethyl acetate, using 200 mL of sample and 500 mL of ethyl acetate at 77 °C for 8 h, reported HYT and TYR concentrations of 776.53 mg/kg FW and 80.62 mg/kg FW, respectively, together with a TPC of 1.74 mg GAE/g FW [71]. Under the same conditions, using OP from the same harvest season and variety, the same authors reported antioxidant activity values of DPPH IC50 = 1.06 mg/mL and ABTS activity, expressed as TRE antioxidant capacity (TEAC) = 0.42 mg/mL [42]. Using the same extraction conditions, Rubio-Senent et al. [43] evaluated the effect of pH on extraction performance. Acidification to pH 2.5 resulted in a TPC of approximately 14.2 g GAE/kg of FW, whereas extraction at pH 4.5 yielded around 11.5 g GAE/kg FW. However, a greater amount of individual phenolic compounds was identified and quantified by HPLC with detectors like DAD in the extract obtained at pH 4.5 (7.4 g at pH 4.5 vs. 5.9 g phenolics/kg of FW at pH 2.5). These findings indicate that pH significantly influences phenolic compound extraction, likely due to differences in the solubility and stability of individual phenolics. These variations affect both the TPC and the phenolic profile of the extracts.

##### Green Extraction Technologies Using Conventional Solvent

- Microwave-Assisted Extraction

MAE is based on the principle that microwave irradiation increases the temperature within the system and enhances solvent diffusion into the solid matrix. This process affects the moisture content of the material, causing rapid evaporation and generating substantial internal pressure within the cell walls, which leads to cell swelling and rupture, thereby facilitating the release of the target organic compounds [20,54].

Different extraction conditions have been reported for MAE, ranging from microwave powers of 100 W [73,74] to 850 W [52]. Although some variations have been observed, microwave power alone generally does not have a significant impact on TPC, antioxidant activity, or the concentration of individual phenolic compounds (Table 2). For example, Marđokić et al. [73] evaluated TPC and antioxidant activity, expressed as GAE and acid ascorbic equivalent (AAE), respectively. Under optimal MAE conditions using 800 W for 180 s, 52.7% ethanol, and a solid-to-liquid ratio of 1:50 (*w*/*v*), values of 14.85 mg GAE/g DW and 10 mg AAE/g DW were obtained. However, similarly high values were also observed at lower powers (15.3 mg GAE/g DW and 9.88 mg AAE/g DW at 450 W; 105 s and a solvent-to-liquid ratio of 1:50 *w*/*v*). Furthermore, another study reported a decrease in antioxidant activity at MAE power levels above 600 W [68]. Collectively, these findings suggest that microwave power is not necessarily the most influential parameter governing extraction performance, as increases in power do not always translate into higher phenol concentrations or greater antioxidant activity. Another parameter evaluated was the solvent type. Macedo et al. [54] reported that, using the same solid-to-liquid ratio and 50% ethanol, a TPC of 240 mg GAE/g DW was obtained under conditions of 35 °C, 12 min and 600 W. However, TPC values decreased with increasing ethanol concentration in the solvent. A similar trend was reported by Habibi et al. [48], who found that ethanol concentrations above 60% significantly reduced HYT content, highlighting solvent composition as a critical factor in MAE.

MAE has enabled the use of a higher solid-to-liquid ratio, which contributes to reduced water consumption and lower effluent generation. Macedo et al. [54] reported the use of water as an extraction solvent at 600 W, while employing a lower solvent volume compared to conventional extraction (1:15 vs. 1:50 *w*/*v*), achieving phenolic extractability comparable to that obtained with conventional ethanol/water extraction systems. In addition, temperature was a key factor influencing the extraction efficiency; with a significant increase observed at 60 °C compared to 40 °C, the extraction yield increased by approximately 30% with the temperature (29.57 mg GAE/g DW at 40 °C and ~22 mg GAE/g DW at 60 °C). This improvement is attributed to viscosity and surface tension decrease at higher temperatures, which facilitates better mass transfer and thus leads to higher yields [20].

Another determining factor is the extraction solvent. The use of methanol/water, ethanol/water, and water has been reported. Xie et al. [27] evaluated different ethanol concentrations and reported the highest HYT concentration at 90% ethanol (56.16 mg/g). Increasing the ethanol concentration to 100% resulted in only a slight increase in HYT content (60.29 mg/g), indicating that extraction efficiency tended to plateau at high ethanol concentrations. The importance of solvent selection in MAE was further demonstrated in another study, where MAE using 50% ethanol and an extraction time of 17 min (5 min ramp and 12 min retention) achieved a TPC of 299 mg GAE/g DW. Under the same solvent conditions, SLE performed in a heating bath for 120 min resulted in a TPC of 240 mg GAE/g DW. These results indicate that MAE can achieve higher TPC values in a considerably shorter extraction time. Furthermore, a comparable TPC value of 289 mg GAE/g DW was obtained when water was used as the extraction solvent. These findings suggest that MAE enables the use of more environmentally friendly solvents without compromising phenolic extraction. It should be noted that the same study also evaluated enzyme-assisted extraction, reporting a further increase in TPC to 342 mg GAE/g DW when tannase (2%) was employed. The aim of this approach is to enhance extraction yield and the production of bioactive compounds by promoting structural modifications of the plant matrix through enzymatic action [54].

- Ultrasound-Assisted Extraction

UAE is based on acoustic cavitation generated by ultrasonic waves, increasing the penetrating power of the solvent and the contact surface between solid and solvent, thereby increasing extraction efficiency. The UAE is one of the simplest and most cost-effective technologies for enhancing industrial scale production [27,75]. UAE has been investigated using several solvents, including ethanol, acetone, water and DES, under different extraction conditions involving temperature, extraction time, and ultrasonic amplitude. TPC, individual phenolic compound concentration and activity antioxidants have been determined under different extraction conditions as indicators of extraction performance and efficiency (Table 2).

Xie et al. [72] conducted a study comparing MAE, SLE and UAE. UAE proved to be the most effective method, resulting in significantly higher concentrations of bioactive compounds: HYT (55.1 mg/g); maslinic acid (381.2 mg/g) and oleanolic acid (29.8 mg/g). These values were significantly higher than those obtained with SLE (49.3 mg/g of HYT; 288.2 mg/g of maslinic acid and 21.5 mg/g of oleanolic acid). However, no significant differences were observed between UAE and MAE. Nevertheless, UAE required a shorter extraction time than MAE (UAE: 3 min vs. MAE: 5 min), which may offer advantages in terms of energy efficiency and process scalability. In addition, in the same study, HYT content decreased significantly at higher ultrasonic frequencies (20 kHz to 60 kHz), unlike maslinic acid and oleanolic acid, whose concentrations appeared to increase, possibly due to their distinct physicochemical properties. The decrease in the concentration of some compounds with increasing ultrasonic frequency may be attributed to the degradation of phenolic compounds at higher frequencies, possibly as a result of hydrogen peroxide formation during the cavitation process.

A positive influence of temperature on UAE performance has been reported, with maximum HYT concentrations observed at around 50 °C [27] and, in another study, a peak antioxidant activity at 56 °C [75]. However, it is important to note that some studies have reported a decline in HYT concentration at higher temperatures [27], while a similar trend has been observed for antioxidant activity above 56 °C [75]. This reduction may be associated with overheating and the excessive pressure generated during ultrasonic cavitation, which can promote the degradation of phenolic compounds and negatively affect antioxidant activity.

Expósito-Almellón et al. [60] evaluated ultrasound amplitude, expressed in µm, as a process variable to improve process scalability, as this parameter is independent of both the sonotrode used and the nominal power of the ultrasonic equipment. The conditions optimized via Response Surface Methodology (RSM) corresponded to an amplitude of 46 µm (approximately 27.6% amplitude), a power density of 25 J mL^−1^, and 100% ethanol as the extraction solvent. Power density was employed as an operational parameter because it is independent of extraction volume and vessel geometry, facilitating process scalability. Under these conditions, OLE and VERB were the major compounds identified, reaching concentrations of 5.0 and 6.6 mg/g dry weight extract (DE), respectively. Furthermore, 12 OLE aglycone isomers were identified, with total concentrations exceeding 15 mg/g DE, while oleacein reached 3.1 mg/g DE.

Conventional single-frequency ultrasound offers several advantages over conventional extraction methods. More recently, Multi-frequency Multimode Modulated (MMM) technology has been developed to overcome some of the limitations of conventional ultrasound systems. MMM minimizes standing-wave effects and improves acoustic field uniformity, resulting in more homogeneous cavitation and more efficient energy transfer, which can enhance extraction performance [90]. A study using MMM reported a higher extraction of phenolic compounds compared with SLE. Using water as the extraction solvent, SLE was performed for 60 min, resulting in a TPC of 256 μg GAE/mL. In contrast, MMM extraction performed with a probe ultrasonic system operating at 160 W for 5 min achieved a TPC of 402 μg GAE/mL. Compared with SLE, MMM achieved higher TPC values using a shorter extraction time [49].

- Supercritical Fluid Extraction

SFE is a green extraction technology that has been widely applied to recover valuable compounds from several raw materials at both laboratory and industrial scales using supercritical fluids as extraction solvents. A pure compound is in a supercritical state when its temperature and pressure exceed its critical values. Carbon dioxide (CO_2_) is the most commonly used supercritical fluid in the food-related extraction processes due to its low cost, non-toxicity, chemical inertness, and relatively low critical pressure and temperature (7.39 MPa and 31.1 °C, respectively) [21]. Under supercritical conditions, CO_2_ exhibits gas-like diffusivity and liquid-like density, providing excellent penetration into solid matrices and enhanced solvation properties. However, because of its relatively low polarity, the extraction of phenolic compounds generally requires the addition of polar co-solvents, such as ethanol and water, to improve their solubility and extraction efficiency (Figure S2). These characteristics enhance mass transfer and facilitate the recovery of target compounds. Furthermore, SFE overcomes several limitations associated with conventional extraction methods, including long extraction times, the use of large amounts of organic solvents, and the degradation of bioactive compounds caused by high extraction temperatures [77].

Caballero et al. [76] reported higher HYT concentrations using SFE (1.25 mg/g) compared to conventional extraction (1.02 mg/g), employing 60% ethanol as the extraction solvent. Additionally, SFE resulted in slightly higher TPC values (14.02 mg GAE/g vs. 12.89 mg GAE/g) and improved antioxidant activity (IC50: 69.19 µg/mL vs. 85.33 µg/mL), compared to conventional extraction. The best extraction performance was achieved at high pressure (300 bar), which enabled a considerable reduction in both extraction time and solvent consumption relative to conventional extraction, from 8 h to 1 h and from 20 mL to 3 mL, respectively. However, similar improvements were not observed in other compounds studied (chlorogenic acid, FER, VA, QUER, VAN and CAFF).

Another study compared OP obtained from different olive varieties and reported significantly higher TPC values for the Coratina variety, ranging from 1.487 to 2.073 mg GAE/g extract, compared with the Arbequina variety (0.867 to 1.468 mg GAE/g extract) under extraction conditions of 200 bar and 3.5 h, indicating that olive variety is an important factor influencing phenolic extraction. Among the process variables evaluated, pressure and ethanol concentration exerted the greatest influence on extraction yield, with the lowest percentage yields (PY) observed at 200 bar and 0% ethanol (3.3% PY). This can be explained by the low polarity of supercritical CO_2_, which is unable to efficiently solubilize polar compounds such as phenolic in the absence of a polar co-solvent [21].

- Pressurized Liquid Extraction

PLE is considered an advanced technology that employs liquid solvents at elevated temperatures and pressures, thereby enhancing extraction efficiency compared with techniques performed at room temperature and atmospheric pressure. Under high-pressure conditions, the solvent remains in the liquid state above its normal boiling point, enhancing both solubility and mass transfer properties (Figure S2). This technique offers several advantages over other extraction methods, including improved selectivity, shorter extraction times, and lower consumption of organic solvents. Moreover, it is an effective technique both at the laboratory scale and in agro-industrial processes [22,91,92].

The PLE system consists of a solvent reservoir connected to a high-pressure pump. The pump delivers the solvent into extraction cells located within a heating system and subsequently carries the extract to collection vials. Within the extraction cells, which are equipped with filters at the outlet, the sample is brought into contact with the solvent either under static conditions at a fixed volume or under continuous flow, thereby eliminating the need for a separate filtration step [92].

Katsinas et al. [24] reported a fivefold increase in HYT concentration (from 1.79 mg/g to 9.5 mg/g) and a threefold increase in TYR concentration (from 1.78 mg/g to 5.3 mg/g) using PLE under optimized conditions established through a circumscribed central composite design (184 °C, 90% ethanol, a sample-to-solvent ratio of 1:1.25 (*w*/*v*), and 20 min), compared with SLE (70 °C, 50% ethanol, a sample-to-solvent ratio of 1:2 (*w*/*v*) and 60 min). Under these conditions, TPC nearly doubled, reaching 340 mg GAE/g compared with 180 mg GAE/g obtained by SLE. Moreover, the extraction time was significantly reduced from 60 to 20 min. Similar results were reported by Cea et al. [22], who identified optimal extraction conditions using RSM at 136.5 °C and 52.3% ethanol. Under these conditions, the total concentration of phenolic compounds (sum of 20 phenolic compounds) was nearly sixfold higher than that obtained by SLE performed using 80% ethanol, an extraction time of 200 min, and a solid-to-solvent ratio of 1:4 *w*/*v* (1659 and 281.7 mg/kg DW, respectively). This significant increase may be attributed to the elevated temperature and pressure applied during the extraction process, which enhances mass transfer and improves the extraction of phenolic compounds.

Various studies have reported that the use of approximately 50% ethanol enhances the extraction of phenolic compounds. OLE and TYR are generally more soluble in organic solvents than in water, which explains their higher extraction under hydroalcoholic conditions. However, when 100% water is used as the extraction solvent, higher HYT extraction is achieved, reflecting its greater polarity and water solubility [22,24,45].

- High Hydrostatic Pressure Extraction

HHPAE is an emerging non-thermal extraction technology that relies on the application of high pressure (in order of MPa) as a driving force to enhance the solubility and mass transfer of target compounds into the solvent within a short period of time. The applied pressure causes air trapped in the vacuoles of plant cells to leak, disrupting the cell membrane and facilitating contact between the solvent and intracellular compounds (Figure S2). This process may alter the conformation or denature cell membrane proteins, thereby increasing the accessibility of phenolic compounds for extraction [93,94].

HHPAE has been studied using organic solvents. Tsevdou et al. [68] reported improved extraction yields using 40% methanol at 650 MPa for 1 min and a solid-to-liquid 0 ratio of 1:10 (*w*/*v*). Under these conditions, TPC and HYT values of 10.1 g GAE/kg and 0.9 g/kg DW, respectively, were obtained, while higher methanol concentrations led to a decrease in TPC (HYT concentration not reported). The same study reported that increasing the solid-to-liquid ratio to 1:30 (*w*/*v*) improved extraction under atmospheric conditions. However, no additional benefits were observed under HHPAE, indicating that high pressure enhances cell permeability and mass transfer sufficiently to achieve efficient phenolic extraction even at lower solvent volumes.

- Ohmic Heating Assisted Extraction

OHE is a green technology based on the direct conversion of electrical energy into thermal energy within food matrices, enabling rapid and uniform volumetric heating. OHE systems operate according to the electrical resistance of conductive materials to the flow of electric current. When an electric current passes through the material, electrical energy is converted directly into heat, resulting in more uniform heating than that achieved by conventional methods (Figure S2). Additionally, OHE provides faster heating rates, lower energy consumption, and improved product quality compared with conventional heating systems [95,96,97].

Quero et al. [30] investigated OHE and reported the highest TPC value of 17.67 mg GAE/g DW when using 50% ethanol at 83 °C for 30 min, compared with 16.89 mg GAE/g DW obtained through conventional heating under the same temperature, solvent and extraction time conditions using a water bath with agitation at 170 rpm. Significant differences were observed in the antioxidant activity, with DPPH and FRAP values reaching 3.82 µmol TRE/g DW and 150.16 µmol Fe(II)/g DW, respectively, compared with 3.56 µmol TRE/g DW and 130.34 µmol Fe(II)/g DW obtained by conventional heating under the same conditions. This improvement is consistent with a 24% increase in total identified phenolic compounds when using OHE with a hydroethanolic solvent, which also resulted in a 15% enhancement in FRAP values. The superior performance of OHE can be attributed to its ability to promote rapid, homogeneous, and selective extraction through electroporation-induced cell membrane disruption, facilitating the release of intracellular compounds and leading to higher extraction yields compared with conventional methods.

- High Pressure, High Temperature Extraction

HPHT reactors are commonly used in enzymatic reactions, molecular synthesis, and microbial inactivation processes. These systems consist of sealed vessels in which the sample matrix is maintained in contact with the solvent under controlled high-temperature and high-pressure conditions. High temperatures reduce the viscosity of liquid solvents, thereby improving their penetration into the matrix and enhancing the extraction efficiency of compounds (Figure S2). Pressurization prevents solvent boiling at elevated temperatures, ensuring that the solvent remains in the liquid phase and in continuous contact with the sample throughout the extraction process, thereby enhancing extraction efficiency [77,98].

HPHT extractions using methanol and 50% ethanol have been reported under similar conditions: sample to solvent ratio 1:10 (*w*/*v*), an extraction time of 90 min and temperature of 180 °C. Methanol extraction yielded a TPC of 45.2 mg CAE/g DW, with OLE as the predominant compound (1406 mg/100 g DW). No significant increase in TPC was observed with increasing extraction time at 100–150 °C; however, higher temperatures (180 °C) enhanced extraction efficiency up to 90 min, followed by a decline, likely due to the thermal degradation of phenolic compounds during prolonged extraction times. On the other hand, extraction with 50% ethanol resulted in a TPC of 57.7 mg CAE/g DW. Although both studies used the same OP variety (Taggiasca), direct comparison is limited due to potential differences in harvest conditions. Nevertheless, considering the polarity of the predominant compound (OLE), these results suggest that hydroethanolic mixtures may enhance phenolic extraction efficiency under HPHT conditions [25,77].

- Pulsed Electric Fields Assisted Extraction

PEF is a non-thermal and non-conventional extraction technique. It is based on exposing cells to high-strength electric fields, which affect the permeability of the cell envelope, including the cell membrane and cell wall. PEF is commonly associated with electroporation (Figure S2). The main advantages of this technology over conventional extraction methods include improved extraction yield, reduced processing time and, consequently, lower intensity of conventional extraction parameters [68,79,99].

PEF treatments were carried out directly in the extraction solvent by applying bipolar, nearly rectangular pulses of 15 μs at an electric field strength of 4.5 kV/cm. PEF significantly enhanced the extraction of phenolic compounds, with extraction time being the most influential factor. Treated samples outperformed untreated controls after both 1 h and 24 h of extraction. Specifically, the application of 5000 pulses increased TPC from 9.8 to 15.3 g GAE/kg after 1 h of extraction. After 24 h, TPC further increased to 18.6 g GAE/kg, compared with 15.7 g GAE/kg in untreated samples. These results suggest that, under the conditions evaluated, extraction time appeared to have a greater influence on phenolic extraction than the intensity of the PEF treatment, although PEF contributed to enhanced extraction efficiency at both extraction times [68]. It should be noted that studies on PEF-assisted extraction of OP remain scarce.

##### Deep Eutectic Solvent-Based Extraction Approaches for Phenolic Extraction from Olive Pomace

To improve the sustainability of extraction processes, increasing research attention has been directed toward the use of DES as alternatives to conventional organic solvents. These solvents have emerged as promising green extraction media due to their low toxicity, biodegradability, low volatility, ease of preparation, and relatively low cost. In addition, DES can be tailored by combining different components, allowing their physicochemical properties to be adjusted according to the characteristics of the target compounds and extraction process [20].

DES are composed of a mixture of two inexpensive and safe components: a hydrogen bond acceptor and a hydrogen bond donor, resulting in a solvent with a melting point lower than that of its individual constituents. This approach seeks to improve the extraction efficiency of a wide range of polar and non-polar compounds [100]. DES are typically classified according to the nature of their components and intermolecular interactions. The most common DES systems are based on quaternary ammonium salts, such as choline chloride, combined with compounds including urea, sugars, or organic acids. A particularly important subgroup is that of natural DES (NADES), which are composed entirely of naturally occurring substances such as amino acids, organic acids, sugars, and choline derivatives. Due to their biocompatibility, low toxicity, and renewable origin, NADES have attracted considerable interest for the extraction of bioactive compounds [101].

A study employing a NADES composed of choline chloride: citric acid (1:2 molar ratio with 20% water) reported a TPC of 34.08 mg GAE/g DW under extraction conditions of 12,000 rpm, 30 min and 60 °C. Increasing the agitation speed from 4000 to 12,000 rpm enhanced phenolic extraction by up to 35%, indicating that agitation speed is an important parameter affecting phenolic recovery. In the same study, the effect of extraction temperature was also evaluated. Increasing the temperature from 40 °C to 60 °C resulted in a rise in TPC from 22.38 to 34.08 mg GAE/g DW, respectively. Similar to what has been observed in SLE, increasing the extraction temperature appears to enhance mass transfer and improve phenolic extraction when DES systems are employed. Under these optimum conditions, concentrations of 12.86 mg/g DW of OLE, 3.37 mg/g DW of HYT, 1.71 mg/g DW of RUT, and 0.12 mg/g DW of LUT were obtained. These concentrations were significantly higher than those obtained using 70% ethanol under the same extraction conditions, where concentrations of 6.34 mg/g DW of OLE, 0.32 mg/g DW of HYT, 0.36 mg/g DW of RUT, and 0.06 mg/g DW of LUT were obtained, while CAFF was not detected. These results highlight the potential of NADES to enhance phenolic compound extraction compared with conventional solvent systems [20]. Further evidence of the influence of temperature was provided by Fernández-Prior et al. [50], who investigated the effect of temperature on the extraction of phenolic compounds using a DES composed of choline chloride: glycolic acid:oxalic acid (1:1.7:0.3 molar ratio). Their most notable finding was that solubilization at 120 °C was higher than at 180 °C, indicating that DES systems can facilitate the extraction of comparable or even greater amounts of bioactive compounds at lower temperatures. Among the temperatures assessed, 120 °C resulted in a TPC of 28.69 mg/g DW using a 1:1 (*w*/*v*) ratio (DES: sample), outperforming both the other solvents evaluated and the higher-temperature condition (180 °C). Excessively high temperatures may lead to the degradation of thermolabile compounds, thereby reducing the TPC.

A more recent study by Carmona et al. [57] also highlighted the high extraction efficiency achieved using a NADES composed of citric acid and fructose (1:1, with 19% water). A concentration of 3988.74 mg/kg FW of phenolic compounds was reported, corresponding to the sum of the individual concentrations of 3,4-dihydroxyphenylglycol (3,4-DHPG), HYT glucoside, HYT, TYR glucoside (salidroside), VERB, the dialdehydic form of decarboxymethyl oleuropein aglycone (3,4-DHPEA-EDA), caffeoyl 6′-secologanoside, and comselogoside. These values were significantly higher than the 2700 mg/kg obtained using 50% methanol under comparable extraction conditions, suggesting the potential of NADES to improve the extraction of phenolic compounds from OP [57].

The influence of water content (25–50%) in DES has also been investigated, as water content can significantly affect the physicochemical properties and extraction performance of NADES. Excessive water can progressively weaken the hydrogen-bond donor–acceptor interactions responsible for DES formation. Nevertheless, water plays a key role in reducing solvent viscosity and density, thereby enhancing mass transfer from plant matrices while lowering extraction costs. Among the NADES systems assessed, glucose: lactic acid (1:5 molar ratio) containing 25% water exhibited the best extraction performance and cost-effectiveness. Under these conditions, significantly higher TPC and FRAP values were obtained (1349.56 mg GAE/100 g FW and 10,603.37 µmol FSE/100 g FW, respectively) compared with those obtained by SLE using 50% ethanol (1071.91 mg GAE/100 g FW and 8765.96 µmol FSE/100 g FW, respectively). However, for the NADES system composed of choline chloride: urea (1:2 molar ratio), which also showed good extraction performance, increasing the water content from 25% to 50% did not compromise extraction efficiency, while reducing solvent viscosity and processing costs [58].

Due to the promising extraction performance of DES, they have been investigated in combination with environmentally friendly technologies to maximize the extraction of bioactive compounds. In this context, DES have been evaluated in combination with several green extraction technologies, including MAE, UAE, and HHPAE.

For MAE, regarding solvent selection, NADES have been investigated as sustainable alternatives to conventional organic solvents. Among the DES formulations evaluated, choline chloride: lactic acid (1:2 molar ratio; 20% water) was selected for MAE. Under extraction conditions of 200 W, 30 min, and a solid-to-liquid ratio of 1:12.5 (*w*/*v*), HYT and TPC values of 0.89 mg/g DW and 29.57 mg GAE/g DW, respectively, were reported. These values represent a nearly threefold increase in HYT concentration compared with extraction using 70% ethanol, which yielded 0.31 mg/g DW of HYT and 10.61 mg GAE/g DW of TPC. These findings highlight the potential of NADES as a complementary and sustainable alternative for enhancing the extraction efficiency of phenolic compounds from OP [20].

In the same study, the authors also evaluated NADES in combination with UAE. The optimal UAE conditions consisted of choline chloride: citric acid (1:2 molar ratio; 20% water) as the extraction solvent, together with a power of 280 W, a frequency of 60 kHz, and a temperature of 60 °C. Under these conditions, a TPC of 20.14 mg GAE/g DW and an antioxidant activity (DPPH IC50) of 20.69 g DW/g DPPH were obtained. These values were higher than those obtained with water extraction (TPC: 10.71 mg GAE/g DW; DPPH IC50: 30.35 g DW/g DPPH). Compared with 70% ethanol extraction, TPC was slightly higher when DES was used, whereas antioxidant activity was greater with 70% ethanol (IC50: 16.81 g DW/g DPPH). Additionally, regarding the sum of individual phenolic compounds quantified by HPLC-DAD, extracts obtained with DES showed higher concentrations than those extracted with either ethanol or water. However, comparison with MAE revealed no significant differences in TPC or antioxidant activity (DPPH IC50), although UAE resulted in a slightly higher HYT concentration (1.05 vs. 0.89 mg/g DW). These findings may represent an opportunity for industrial implementation, as UAE offers advantages in terms of lower capital investment, operational simplicity, and scalability compared with MAE.

A more recent study combining UAE and DES evaluated the use of β-cyclodextrin, a glucose-based oligosaccharide derived from the enzymatic degradation of starch, as an extraction enhancer due to its ability to selectively form inclusion complexes with target analytes. The combined use of β-cyclodextrin and a DES composed of ammonium acetate and lactic acid (1:7 molar ratio with 35% water) was investigated, with an optimum β-cyclodextrin concentration of 1.8%. Lower concentrations (0.7%) resulted in reduced extraction yields, likely due to insufficient complex formation, whereas higher concentrations negatively affected extraction efficiency, possibly because of increased solvent viscosity. The optimum extraction conditions were 43 °C, 22 min, and a solid-to-liquid ratio of 1 g/48 mL, under which a TPC of 56.98 mg GAE/g DW was obtained. This value was 26% higher than that obtained using the DES alone and approximately 104% higher than conventional water- or ethanol-based extractions [59].

Additionally, a study employing HHPAE using a NADES composed of choline chloride: lactic acid (1:2 molar ratio; 20% water) reported up to tenfold higher concentrations of key phenolic compounds compared with water extraction, including OLE (1.94 vs. 0.19 mg/g DW), HYT (2.57 vs. 0.25 mg/g DW) and CAFF (0.01 vs. 0.001 mg/g DW), under the same pressure and extraction time conditions. In the case of extraction using 70% ethanol, under the same conditions, increases of approximately sixfold for HYT and tenfold for CAFF were observed. However, OLE showed a different behavior, with significantly higher concentrations in 70% ethanol (4.44 mg/g DW) than in DES-based extracts (1.94 mg/g DW), suggesting a greater affinity of this compound for the hydroethanolic solvent system [20]. Regarding pressure, the same study reported improved phenolic extraction at 600 MPa for 10 min, achieving a TPC of 25.96 mg GAE/g DW. In contrast, under the same extraction time, but at 300 MPa, TPC values were approximately 15 mg GAE/g DW. Similarly, antioxidant activity, expressed as DPPH IC50, improved at 600 MPa, reaching 15.67 g DW/g DPPH, whereas values higher than 25 g DW/g DPPH were obtained at 300 MPa for 10 min. These results indicate that increasing pressure can enhance both phenolic extraction and antioxidant activity.

Recent studies have increasingly explored the integration of DES with advanced extraction technologies, including PLE, MAE, and UAE. These hybrid approaches seek to improve extraction efficiency while maintaining the sustainability advantages associated with green solvents [102].

Although DES have demonstrated significant potential for improving phenolic compound extraction, their industrial implementation remains subject to economic and environmental considerations. In general, DES formulations tend to be more expensive than conventional solvents such as ethanol due to the cost of their constituents and, in some cases, the additional processing steps required for their synthesis [58]. Furthermore, the economic feasibility of DES-based extraction depends not only on solvent preparation costs but also on solvent recovery, recycling, and process scalability.

Recent reviews have highlighted the potential of DES as sustainable extraction media due to their low toxicity, tunable physicochemical properties, broad solubilization capacity, and ability to enhance extraction efficiency compared with conventional solvents. Nevertheless, their environmental performance is highly dependent on the chemical nature of the hydrogen-bond donor and acceptor components, as well as on solvent preparation, recovery, and recycling strategies [103]. Some studies have reported that DES composed of naturally derived constituents exhibit favorable biodegradability profiles, whereas others may present less favorable sustainability profiles. For instance, life-cycle assessment studies have shown that DES based on choline chloride: urea may generate lower environmental impacts than conventional extraction solvents such as dichloromethane and ethyl acetate, although not necessarily lower than methanol or ethanol. In contrast, certain DES formulations based on choline chloride and citric acid have been associated with higher environmental burdens, partly due to water consumption and carbon dioxide emissions related to citric acid production [104].

Overall, although DES represent promising alternatives for sustainable extraction, their environmental and economic performance should be evaluated on a case-by-case basis. Further studies addressing solvent recyclability, life-cycle impacts, biodegradability, and large-scale process economics are required to support the industrial implementation of DES-based extraction systems [105].

#### 2.2.3. Comparative Evaluation of Extraction Technologies

**Figure 3:**
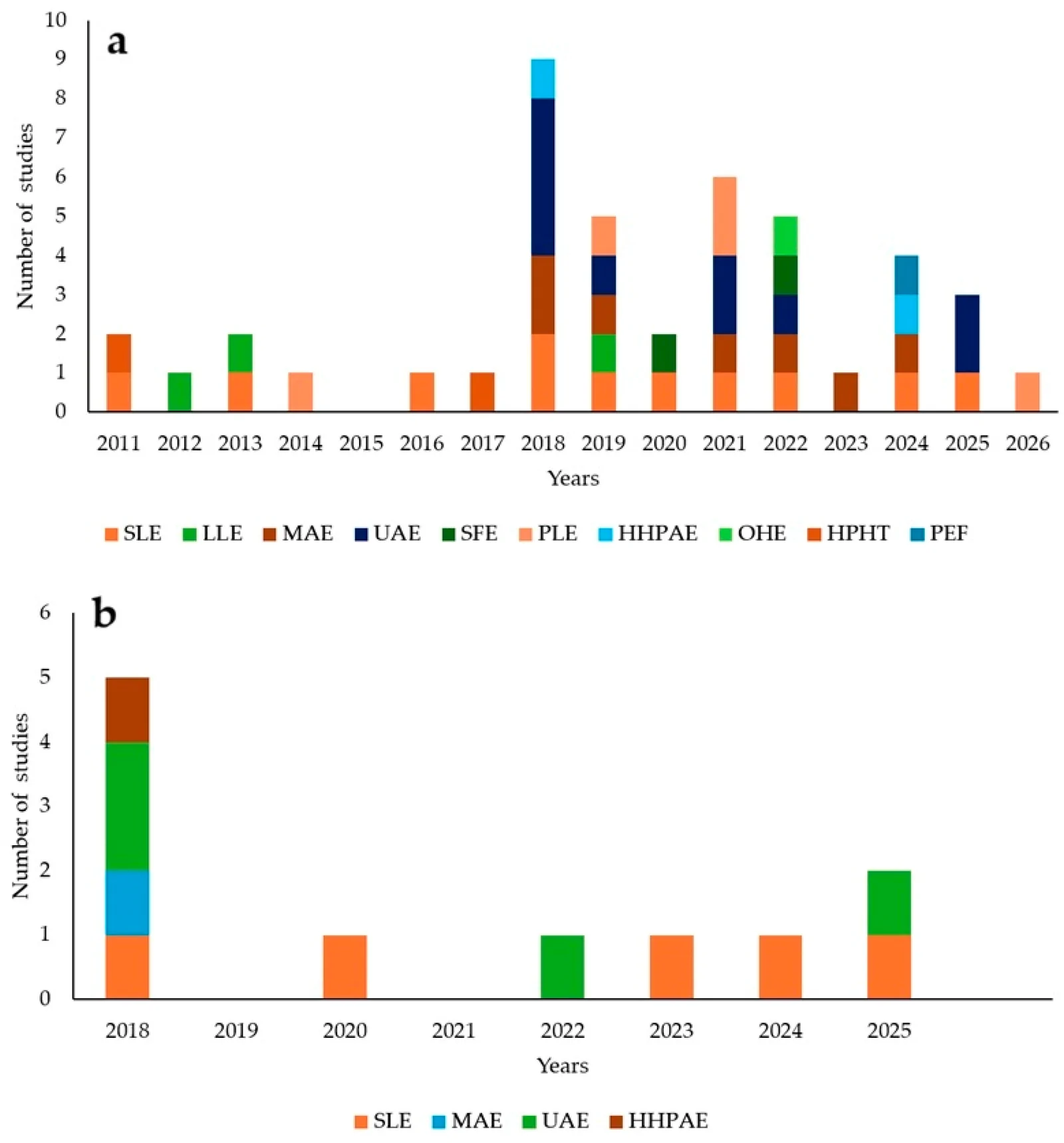
Temporal trends in the use of extraction technologies for phenolic compound recovery from olive pomace. (**a**) Conventional and green extraction technologies. (**b**) DES-based extraction approaches. SLE: solid–liquid extraction; LLE: liquid–liquid extraction; MAE: microwave-assisted extraction; UAE: ultrasound-assisted extraction; SFE: supercritical fluid extraction; PLE: pressurized liquid extraction; HHPAE: high hydrostatic pressure-assisted extraction; OHE: ohmic heating extraction; HPHT: high-pressure and high-temperature extraction; PEF: pulsed electric field-assisted extraction.

In the pursuit of more efficient extraction strategies, a significant increase in the development and adoption of greener extraction approaches has been observed since 2018. These emerging techniques aim to provide innovative alternatives that reduce solvent consumption, shorten extraction times, and enhance overall sustainability (Figure 3a,b).

A marked increase in studies employing UAE was observed in 2018; however, its prevalence subsequently declined as emerging technologies gained attention. Although PLE was first reported for OP in 2014, its application became more widespread from 2019 onwards. Similarly, studies investigating SFE for the recovery of phenolic compounds from OP began to emerge in 2020. More recently, OHE and PEF were reported in 2022 and 2024, respectively, and to date remain relatively unexplored technologies for OP valorization. HHPAE has also attracted interest, with investigations reported in 2018 and again in 2024. In contrast, conventional and well-established techniques, including SLE, MAE, and UAE, using both conventional solvents and DES, have remained the most extensively investigated since 2018. This continued interest may be attributed to their lower equipment costs, operational simplicity, and scalability potential (Figure 3a). Furthermore, the use of DES has attracted considerable attention, particularly in 2018, when the highest number of studies evaluating DES in combination with SLE, UAE, MAE, and HHPAE was reported. More recently, DES have also been combined with β-cyclodextrin to further enhance phenolic compound extraction. These developments highlight the growing interest in DES as sustainable extraction media and their potential integration with other extraction technologies (Figure 3b).

Although direct comparisons among studies are limited by differences in extraction methods, olive cultivars, and analytical approaches (Table 2), several factors influencing extraction performance can be identified. In conventional extraction methods, agitation speed and solvent composition play important roles, with ethanol–water mixtures generally providing higher antioxidant activity and enhanced extraction of major phenolic compounds such as HYT, OLE, and TYR. Moreover, ethanol–water mixtures are considered more sustainable than many conventional organic solvents due to the low toxicity, renewability, and food-grade status of ethanol, while still achieving high extraction performance DES-based extraction has demonstrated superior phenolic extraction compared with conventional organic solvents, although long extraction times and relatively high solvent consumption may limit its overall efficiency [20,24,69].

Green extraction technologies have generally improved extraction efficiency while reducing solvent consumption and processing time. Among them, MAE frequently achieves the highest phenol extraction, whereas UAE offers a more cost-effective, energy-efficient, and scalable alternative despite generally lower extraction performance [27,73]. PLE and HHPAE have also shown promising results, achieving high phenolic concentrations at relatively short extraction times (approximately 20 min and 1–10 min, respectively). In contrast, SFE, OHE, HPHT, and PEF have demonstrated satisfactory extraction performance, although their effectiveness has generally been lower than that reported for MAE, PLE, or HHPAE under the conditions evaluated [106].

When comparing extraction technologies, extraction performance should not be evaluated solely on the basis of phenolic recovery. Parameters such as extraction time, solvent consumption, energy requirements, scalability, and environmental impact should also be considered. For example, MAE frequently provides high phenolic recoveries within short processing times, whereas UAE generally requires longer extraction periods but offers greater operational simplicity and easier scale-up. Similarly, DES-based systems have demonstrated promising extraction performance but often require higher solvent-to-solid ratios and longer extraction times than conventional hydroalcoholic systems. Therefore, the selection of an extraction strategy should be based on a balance between extraction efficiency, process economics, environmental considerations, and industrial feasibility, rather than on extraction yield alone.

In addition to extraction yield and processing time, energy consumption is an important parameter when evaluating the industrial feasibility of green extraction technologies. However, direct comparison of energy efficiency among technologies remains challenging due to substantial differences in the way process intensity is reported. For example, MAE studies employed microwave powers ranging from 220 to 850 W, whereas UAE studies reported powers between 120 and 400 W, often combined with different frequencies, amplitudes, sonotrode dimensions, extraction volumes, and reactor configurations. Only one recent UAE study reported a specific energy input value (25 J mL^−1^), a parameter considered less dependent on instrument characteristics. Therefore, the lack of standardized reporting prevents a robust comparison of energy efficiency across studies and hinders techno-economic assessments of extraction technologies.

Despite their potential, technologies such as SFE, PLE, HHPAE, OHE, HPHT, and PEF remain insufficiently studied, making systematic comparisons difficult. Furthermore, several of these technologies require substantial capital investment and still face challenges related to industrial-scale implementation. Overall, UAE appears particularly attractive for large-scale applications due to its operational simplicity, relatively low investment costs, and scalability potential. Regardless of the extraction technology employed, process variables such as temperature, solvent composition, and extraction enhancers, including DES, β-cyclodextrin, or enzymatic treatments, should be carefully optimized to maximize the extraction of bioactive compounds.

## 3. Biological Activities and Health-Promoting Effects of Olive Pomace Extracts

Several studies have investigated the toxicological and biological activities of OP extracts, particularly through the assessment of cell viability in both cancerous and healthy cell lines. The available evidence consistently highlights antitumoral, antioxidant, and antiproliferative effects, with OP extracts and their bioactive compounds exhibiting selective cytotoxicity toward cancer cells while exerting limited effects on healthy cells. This selective behavior supports the safety profile of OP-derived compounds and has been reported by Bermúdez-Oria et al. [107], Ferreira et al. [108] Radić et al. [109]. These bioactivities have been primarily attributed to HYT, OLE, TYR, and VERB, which occur at high concentrations in OP extracts.

The antioxidant and cytoprotective properties of OP extracts have also been widely documented. Hydroethanolic OP extracts protected non-tumorigenic human hepatoma cells subjected to oxidative stress, enhancing cellular redox status through reducing activity and radical-scavenging mechanisms [109,110]. Bermúdez-Oria et al. [107] reported reduced intracellular levels of reactive oxygen species (ROS) and nitrite (NO) in murine peritoneal macrophages, while antioxidant protection was also observed in intestinal cells.

Several studies have further confirmed the therapeutic potential of OP extracts and their major phenolic compounds, particularly HYT, OLE, TYR, and VERB. In this context, Anter et al. [111] investigated the cytotoxicity of OP and its major phenolic compounds (HYT, TYR, and VERB), reporting no genotoxic effects. Moreover, both OP extracts and HYT exhibited antiproliferative and pro-apoptotic activity against a human promyelocytic leukemia cell line. In another study, OLE demonstrated selective cytotoxicity toward pancreatic cancer cells, whereas HYT exerted selective effects on both pancreatic cancer cells and non-tumorigenic pancreatic cells [112]. These findings suggest that the therapeutic properties of OP extracts are closely associated with the biological activity of their major phenolic constituents.

Additional evidence supports the anticancer, anti-inflammatory, and neuroprotective potential of OP-derived phenolics. Antineuroinflammatory activity has been demonstrated in microglial cells, the brain’s resident immune cells [113]. Methanolic extracts inhibited cancer cell proliferation, particularly in breast cancer cells, while exerting protective effects on non-tumorigenic pancreatic cells [108]. Likewise, aqueous extracts showed antiproliferative and anti-inflammatory activity against colon carcinoma cells and human corneal epithelial cells [24,44,107,114]. Furthermore, Camilleri et al. [115] found that methanolic OP extract encapsulated in liposomes decreased cell viability.

Additional health-promoting effects have also been reported. Palmieri et al. [116] reported cardioprotective activity by preventing anoxia-induced damage in human endothelial cells. Moreover, methanolic and ethanolic OP extracts obtained using green extraction techniques, such as OHE and UAE, have demonstrated anticancer and antioxidant protective effects in intestinal cells [30,55]. Similarly, OP extracts produced through a patented pressing method significantly reduced cell proliferation in breast, pancreatic, and colorectal cancer cell lines [108]. Collectively, these findings demonstrate that OP extracts obtained through different extraction approaches retain significant bioactive potential. Importantly, the observed biological effects are closely linked to the concentration and composition of phenolic compounds present in the extracts [55,107,115].

In addition to their antioxidant and anti-inflammatory properties, several studies have also reported antimicrobial activity of OP extracts against foodborne and pathogenic microorganisms, suggesting their potential application as natural preservatives and functional ingredients in food systems [61,117].

Collectively, these studies demonstrate that OP extracts exert biological effects in a wide range of experimental models, including intestinal, hepatic, endothelial, neuronal, immune, and cancer cells. The most consistently reported activities include antioxidant, anti-inflammatory, cardioprotective, and selective antiproliferative effects, highlighting the potential of OP-derived phenolics as functional ingredients with health-promoting properties.

## 4. Food Applications of Olive Pomace and Its Extracts

Due to its potential health benefits, several studies have explored the incorporation of OP or OP extract into food products with the aim of improving product stability while using foods as carriers of bioactive compounds. Such approaches not only enhance the nutritional profile of the final food but may also facilitate the delivery of health-promoting compounds to consumers. Applications of OP include fermented cakes and bakery products enriched with milled OP [118,119], fish burgers [120], granola, bread, pasta, cookies, and extruded rice oat foods, all showing improved nutritional profiles [120,121,122,123,124,125,126].

OP and fresh tomato pomace extracts have been used to enrich an olive paste dip, resulting in a product with high nutritional value due to its carotenoid and phenolic content [127]. Fortification with oil-in-water (O/W) emulsions has also been evaluated in beverages, showing good bioaccessibility in vitro [128]. OP extract has been applied as a natural antioxidant in edible vegetable oils, offering an alternative to synthetic antioxidants [41,61,129,130], and has been used to fortify fermented dairy products without affecting the fermentation process, demonstrating the stability of phenols throughout production. Sensory analysis of this product revealed favorable organoleptic properties, supporting its potential as a functional food ingredient [131]. Furthermore, Detopoulou et al. [132] evaluated yogurt fortified with a polar lipid extract of OP to investigate its effect on platelet-activating factor (PAF), a pro-inflammatory lipid mediator involved in atherosclerosis. The fortified yogurt demonstrated favorable modulation of PAF, suggesting its potential as a functional food for cardiovascular disease prevention.

Encapsulation techniques have been employed to enrich food products with OP extract. Bioaccessibility studies indicate that encapsulated extracts generally exhibit higher bioaccessibility than non-encapsulated counterparts, likely due to the protective effect provided by the encapsulation matrix [109,133,134]. Microencapsulated OP extract added to fresh pasta enhanced phenol content and nutritional quality, with positive sensory evaluation by consumers [135]. Additionally, Katsouli et al. [128] reported that emulsions used for functional beverage fortification showed greater stability with smaller droplet sizes and narrower size distribution, as well as higher oil viscosity, which reduced coalescence. Simulated gastrointestinal studies demonstrated high bioaccessibility of phenolic compounds, indicating effective release and solubilization during digestion, along with good storage stability, supporting the use of emulsions as food ingredients.

These examples demonstrate the successful incorporation of OP-derived ingredients into commercially relevant food matrices, including dairy products, pasta, bakery foods, snacks, fish-based products, and functional beverages. In most cases, OP addition improved the nutritional profile and antioxidant properties of the products while maintaining acceptable technological and sensory characteristics.

Based on the reviewed literature, OP and OP extract exhibit considerable potential as food ingredients due to their safety, bioactivity, and potential health-promoting effects. Encapsulation strategies may further enhance their bioaccessibility and protect bioactive compounds from degradation during food processing, storage, and gastrointestinal digestion.

## 5. Safety, Regulatory, and Commercialization Aspects of Olive Pomace-Derived Ingredients

Despite the promising biological activities and food applications reported for OP-derived ingredients, several aspects should be considered before their large-scale commercialization. Safety assessments conducted on OP extracts and their major phenolic constituents have generally demonstrated low toxicity and the absence of genotoxic effects under the evaluated conditions. However, the safety profile may vary depending on the extraction technology employed, solvent residues, concentration factors, and the presence of process-derived contaminants.

From a regulatory perspective, the commercialization of OP-derived ingredients is subject to different legal requirements depending on the target market. In the European Union, certain phenolic-rich extracts and hydroxytyrosol-enriched preparations may fall within the scope of the Novel Food Regulation (EU) 2015/2283 when a sufficient history of safe consumption cannot be demonstrated [136]. Similar pre-market safety assessment frameworks are applied in several jurisdictions worldwide. In Australia and New Zealand, novel food ingredients are regulated under Standard 1.5.1 of the Food Standards Code and require a safety assessment prior to commercialization [137]. In Canada, novel foods are subject to mandatory pre-market notification and safety assessment by Health Canada, whereas in the United States, market access may depend on food additive approval or Generally Recognized as Safe (GRAS) status [138,139]. Similar regulatory frameworks governing novel and functional ingredients exist in Asian markets, including China and Japan, although authorization procedures and safety requirements differ among jurisdictions. Therefore, the regulatory requirements applicable to OP-derived ingredients vary considerably among jurisdictions and should be carefully evaluated before commercialization.

In addition to regulatory compliance, industrial implementation requires consideration of solvent recovery, residual solvent content, contaminant control, product stability, and compositional standardization. These aspects are essential to ensure consumer safety, regulatory compliance, product quality, and successful market adoption of OP-derived ingredients. Therefore, future research should not only focus on improving extraction performance but also on generating safety, stability, and regulatory data to facilitate commercialization and broader industrial implementation.

## 6. Conclusions and Future Trends

Animal and human studies further support the safety and functional potential of OP. The available evidence suggests that OP-derived phenolics may exert beneficial effects through antioxidant, anti-inflammatory, and metabolic mechanisms, supporting their potential incorporation into functional foods and other value-added applications [80,125,132].

Since 2018, there has been a growing trend toward extracting bioactive compounds from OP using greener technologies. Among the most widely applied approaches is UAE, owing to its low investment cost, operational simplicity, and ability to achieve efficient recovery of phenolic compounds. Furthermore, the studies reviewed have reported promising results using NADES and extraction enhancers such as β-cyclodextrin, which have demonstrated considerable capacity to enhance phenolic compound recovery and overall extraction efficiency under specific processing conditions. More recently, research has increasingly focused on integrating additional green extraction techniques with the aim of improving processing time, extraction efficiency, and overall cost-effectiveness.

The potential use of OP extracts as functional ingredients has also attracted increasing attention. Given their strong in vitro bioactivity, incorporation into food matrices appears highly promising. In this context, encapsulation techniques have been explored as a strategy to improve the organoleptic properties, stability, and bioaccessibility of OP-derived compounds, opening new opportunities for future research and food applications.

In conclusion, OP represents a valuable source of phenolic compounds with significant antioxidant, antimicrobial, and health-promoting properties. Despite substantial advances in extraction technologies, analytical characterization and application development, several challenges still hinder their large-scale utilization. The first major bottleneck is the economic viability of extraction processes, particularly regarding equipment investment, energy consumption, solvent recovery, and purification costs. A second challenge involves maintaining polyphenol stability during processing, storage, and incorporation into final products, as degradation reactions may reduce their functionality and shelf life. A third major challenge is the regulatory framework governing olive-derived phenolic ingredients. Although several jurisdictions, including the European Union, the United States, Canada, and Australia/New Zealand, have established regulatory pathways for novel and functional food ingredients, safety requirements, authorization procedures, and market access conditions differ substantially among regions. Therefore, the commercialization of olive-derived phenolic ingredients requires careful evaluation of the applicable regulatory framework in each target market. Future research should therefore focus not only on improving extraction yields but also on developing cost-effective processes, enhancing polyphenol stability, and facilitating regulatory approval to support the successful valorization of olive residues within a circular bioeconomy framework.

## Figures and Tables

**Table 2:** Comparison of OP Extraction Methods.

Olive Pomace Pretreatment	Solvent Extraction	Ratio Sample/Solvent	Treatment Conditions	Measured Parameter	Ref
**Conventional Solvent-Based Extraction Methods**					
Fat removal with N-hexane (1:5 (*w*/*v*)), 1 h	SLE: Ethanol (pH 2)	1 g/5 mL	Orbital shaker; 180 min; Room temperature	DPPH 59.8% (inhibition); Extraction yield 95.3%	[40]
Drying 40 °C, 3 days until constant weight	SLE: Water	2 g DW/30 mL	Constant agitation; 40 min; Room temperature	TPC 78.2 mg GAE/g DW; DPPH 57.15 µg/mL (IC50); HYT 25.21 mg/g DW; TYR 1.3 mg/g DW; OLE 1.74 mg/g DW; VERB 7.02 mg/g DW	[44]
Extraction with petroleum ether by sonication 15 min at 40 KHz room temperature	SLE: 80% Methanol	450 g/1.5 L	40 °C	TPC 275 mg QE/g FW (Barnea), 156 mg QE/g FW (Arbequina); TFC 56.3 mg QE/g FW (arbequina), 43.8 mg QE/g FW (Frantoio); DPPH (IC50) 27.9 µg/mL (Barnea), 41.1 µg/mL (Arbequina), 48.7 µg/mL (Frantoio); HYT 4.93 µg/mg extract (Barnea) TYR 0.23 µg/mg extract (Arbequina)	[46]
Separation of OP skin from pit	SLE: DMSO	10 g FW/20 mL	Ultra-Turrax; 30 min; Room temperature	Triterpenic acids 120 g/kg (70% maslinic acid) [1 g DW and triturated pomace was mixed in a 10 mL centrifuge tube with 4 mL methanol–ethanol (1:1, *v*/*v*) and vortexed for 1 min]	[36]
Freeze-dried	SLE: Water	1 g/50 mL	600 rpm; 60 min; 40 °C	TPC 256 µg GAE/mL; DPPH ≈ 500 µg TRE/mL; FRAP ≈ 3.5 µmol FSE/mL	[49]
Freeze-dried	SLE: 80% Methanol	5 g/20 mL	120 min; Room temperature	Phenols 282 mg/kg (20 compounds); OLE 22 mg/kg DW; API 12.4 mg/kg DW; LUT 50 mg/kg DW	[22]
Pitted, palletized and fat removal with N-hexane (6.5% moisture)	SLE: Water	15 g/100 mL	150 rpm; 90 min; 55 °C	TPC 38.1 mg GAE/g OP; TFC 71.4 mg RE/g OP; DPPH 22.4 mg TRE/g OP; ABTS 70.7 mg TRE/g OP; FRAP 39.9 mg TRE/g OP	[67]
	SLE: 0.5% Acetic acid			TPC 29.7 mg GAE/g OP; TFC 63.3 mg RE/g OP; DPPH 16.3 mg TRE/g OP; ABTS 57.1 mg TRE/g OP; FRAP 33.9 mg TRE/g OP	
	SLE: 50% Ethanol			TPC 39.5 mg GAE/g OP; TFC 76.3 mg RE/g OP; DPPH 27.9 mg TRE/g OP; ABTS 62.9 mg TRE/g OP; FRAP 41.5 mg TRE/g OP	
	SLE: 20% Ethanol			TPC 34.6 mg GAE/g OP; TFC 67.1 mg RE/g OP; DPPH 22.4 mg TRE/g OP; ABTS 64.2 mg TRE/g OP; FRAP 38.1 mg TRE/g OP	
	SLE: 50% Acetone			TPC 41.6 mg GAE/g OP; TFC 76 mg RE/g OP; DPPH 35.1 mg TRE/g OP; ABTS 63.5 mg TRE/g OP; FRAP 46.2 mg TRE/g OP	
	SLE: Water	10 g/100 mL	150 rpm; 90 min; 85 °C	TPC 44.5 mg GAE/g OP; TFC 114.9 mg RE/g OP; DPPH 36.1 mg TRE/g OP; ABTS 95.4 mg TRE/g OP; FRAP 47.6 mg TRE/g OP; HYT 6.3 mg/g OP	
Freeze-dried and defatting with CO_2_ flow rate was set at 10.5 kg CO_2_/h for an extraction time of 3 h (20 °C and 6 Mpa)	SLE: 50% Ethanol	0.5 g/1 mL	750 rpm; 60 min; 70 °C	TPC 180 mg GAE/g DE; TFC 9 mg CATE/g DE; ORAC 4.6 mmol TRE/g DE; HYT 1.79 mg/g DE; TYR 1.78 mg/g DE; OLE 2.4 mg/g DE	[24]
Drying 60 °C, 48 h.	SLE: 50% Ethanol	2 g/20 mL	170 rpm; 30 min; 83 °C	TPC 16.89 mg GAE/g DW; DPPH 3.56 µmol TRE/g DW; FRAP 130.34 µmol FE(II)/g DW; HYT 33.49 mg/100 g DW; TYR 11.09 mg/100 g DW; OLE 365.05 mg/100 g DW	[30]
	SLE: Water			TPC 12.24 mg GAE/g DW; DPPH 3.36 µmol TRE/g DW; FRAP 80.45 µmol FE(II)/g DW; HYT 31.20 mg/100 g DW; TYR 11.13 mg/100 g DW	
Freeze-dried and milled	SLE: Methanol	1 g/15 mL	Fixed Bed Semi-Batch Extraction; 60 min; 25 °C	TPC 13.7 g GAE/kg DW, 78.1 g GAE/kg DE; TFC 0.8 g/kg DW, 3.9 g/kg DE; HYT 3.5 g/kg DW, 16 g/kg DE; LUT 0.6 g/kg DW, 2.7 g/kg DE	[68]
Crushes, kneading, underwent and centrifugation to extract the oil	SLE: 61.2% Ethanol	0.3 g/15 mL	15,000 rpm; 15.82 min; Room temperature	TPC 60.28 g GAE/kg OP; TFC 20.96 g CE/kg OP; Ortho-diphenols 39.82 g GAE/kg OP	[69]
Freeze-dried	SLE: Water (3 extraction)	1–50 g/100 mL 2–3 remaining solid/50 mL	1500 rpm, 120 min/extraction; 4 °C	TPC 0.29–1.02 kg/m^3^	[70]
	LLE: Ethyl acetate pH 2	50 mL/150 mL	Magnetic stirring; 120 min; 20 °C	TPC 0.5–1.2 g CAE/kg	
20 kg OP underwent hydrothermal treatment at 160 °C for 75 min. The mixture was centrifuged, and 10 L of supernatant was concentrated to 1 L. Lipids were removed by hexane washing (1:0.5 *v*/*v*).	LLE: Ethyl acetate	200 mL/500 mL	Continuous extraction; 480 min; 77 °C reflux	DPPH 1.06 mg/mL (IC50); ABTS 0.42 mg/mL (TEAC); Reducing power 0.42 mg TRE/mL	[42]
20 kg of OP underwent hydrothermal treatment at 160 °C for 60 min. The mixture was centrifuged, and 10 L of supernatant was concentrated to 1 L. Lipids were removed by hexane washing (1:0.5 *v*/*v*).	LLE: Ethyl acetate	200 mL/500 mL	Continuous extraction; 480 min; 77 °C	TPC 1.74 mg GAE/g FW; HYT 776.53 mg/kg FW; TYR 80.62 mg/kg FW; OLE 0.46 mg/kg FW; CAFF 2.65 mg/kg FW; VERB 8.43 mg/kg FW	[71]
20 kg of OP underwent hydrothermal treatment at 160 °C for 60 min. The mixture was centrifuged, and 10 L of supernatant was concentrated to 1 L. Lipids were removed by hexane washing.	LLE: Ethyl acetate pH 4.5 and 2.5	200 mL/500 mL	Continuous extraction; 480 min; 77 °C reflux	TPC 11.5 * g GAE/kg FW (pH 4.5); 14.2 * g GAE/kg FW (pH 2.5); DPPH ≈ 1 mg/mL IC50 (pH 4.5); ≈ 2 mg/mL IC50 (pH 2.5); Reducing Power ≈ 0.5 mg TRE/mL (pH 4.5); ≈ 0.3 mg TRE/mL (pH 2.5); HYT 1462 * mg/kg FW (pH 4.5); 816 * mg/kg FW (pH 2.5); TYR 173 * mg/kg FW (pH 4.5); 92.5 * mg/kg FW (pH 2.5); OLE 13.8 * mg/kg FW (pH 4.5); 8 * mg/kg FW (pH 2.5)	[43]
**Green Extraction Technology**					
**Microwave extraction (MAE)**					
Dried and milled	MAE: 60% Ethanol	0.5 g/5 mL	220 W; 12 min; Room Temperature	HYT 1224.1 * mg/kg; OLE 2495.6 * mg/kg	[48]
	DLLME: 60 µL 1-octanol and 500 µL of ethanol		Orbital shaker; 2 min; Room temperature		
Drying 50 °C, 48 h. Sieved and pulverized	90% Ethanol	1 g/30 mL	2.450.000 kHz/600 W; 5 min; 50 °C	HYT 53.2 mg/g DW; Maslinic acid 356 mg/g DW; Oleanolic acid 26.3 mg/g DW	[72]
Centrifugation	Water with 2% Tannase enzyme	1 g/15 mL	600 W; Ramp 5 min, retention 12 min; 60 °C	TPC 341 mg GAE/g DW; DPPH 60% (inhibition); ABTS 65% (inhibition); HYT 47.5 mg/kg DW; TYR 11 mg/kg DW; CAFF 16.8 mg/kg DW FER 53.1 mg/kg DW	[54]
Drying (6.5% moisture) and milled	Water	12% *w*/*v*	850 W/30 bar; 16 min; 100 °C	TPC 34.5 mg GAE/g OP; FRAP 45.4 mg TRE/g OP; ABTS 98.8 mg TRE/g OP; HYT 5.8 mg/g OP	[52]
Drying 40 °C (5% moisture)	52.7% Ethanol	1 g/50 mL	800 W; 3 min (180 s); Room temperature	TPC 14.85 mg GAE/g DW; FRAP 10.00 mg AAE/g DW	[73]
Drying 40 °C, 24 h and milled	60% Methanol	1 g/10 mL	300 W; 5 min; 50 °C	TPC 11.4 g GAE/kg DW, 132 g GAE/kg DE; TFC 0.7 g/kg DW, 7.5 g/kg DE; HYT 2.7 g/kg DW, 31.5 g/kg DE; LUT 0.4 g/kg DW, 4.3 g/kg DE	[68]
**Ultrasound extraction (UAE)**					
Freeze-dried, milled and sieved. Fat removal with hexane (1:10 *w*/*v*) 3 times	Water	2 g/100 mL	250 W (bath)/Amplitude 100%; 75 min; 30 °C	TPC 19.71 mg GAE/g DW; DPPH 31.23 mg TRE/g DW; CUPRAC 73.54 mg TRE/g DW	[74]
Ground and defatted with petroleum ether	Methanol	3.6% *w*/*v*	200 W/20 kHz (probe 19 mm); 3 min, cycles 0.6 s; 56 °C	TPC 4.04 mg GAE/g; AA 68.9% (β-Carotene)	[75]
Freeze-dried	Water	1 g/50 mL	MMM-technique 160 W/20 KHz (probe); 5 min; Room temperature (up to 75 °C)	TPC 402 µg GAE/mL; DPPH ≈ 1200 µg TRE/mL; FRAP ≈ 7 µmol FSE/mL; HYT 238.42 mg/100 g DW; TYR 9.6 mg/100 g DW	[49]
Drying 50 °C, 48 h. Sieved and pulverized	90% Ethanol	1 g/30 mL	135.6 $\frac{W}{{cm}^{2}}$ /20 kHz (probe 13 mm); 3 min; 50 °C	HYT 55.1 mg/g DW; Maslinic acid 381.2 mg/g DW; Oleanolic acid 29.8 mg/g DW	[72]
Drying (6.5% moisture) and milled	40% Acetone	8.6% *w*/*v*	150 W/40 kHz/70% amplitude (probe 3.17 mm); 12 min; Room temperature (up to 46 °C)	TPC 45.41 mg GAE/g OP; DPPH 35.61 mg TRE/g OP; ABTS 140.67 mg TRE/g OP; FRAP 68.08 mg TRE/g OP; HYT 4.96 mg/g OP	[51]
Freeze-dried (2 days) and then removed from the pit and pulverized	90% Ethanol	1:30 *w*/*v*	120 W/40 kHz; 30 min; Room temperature	Frantoio (Variety): VERB 4.88 g/kg DW; Maslinic acid 1319 mg/kg DW; Oleanolic acid 783 mg/kg DW; Total triterpenoids 2102 mg/kg DW	[55]
Freeze-dried and milled	Ethanol	2 g/20 mL	400 W/24 kHz ≈27.6% amplitude (46 µm)/25 J × mL^−1^; ≈1.83 min; ≈17 °C (≤40 °C)	TPC (obtained as sum of total of identified phenolic compounds) 43 mg/g DE; DPPH 128 mg TRE/g DE; HYT 0.5 mg/g DE; OLE 5 mg/g DE; VERB 6.6 mg/g DE; LUT 2.26 mg/g DE	[60]
**Supercritical Fluid Extraction (SFE)**					
Pellets	CO_2_ with 60% Ethanol	20 g/60 mL	300 bar; 60 min; 50 °C	TPC 14.01 mg GAE/g; DPPH 85.33 µg/mL (IC50); HYT 1.25 mg/g; CAFF 0.05 mg/g; FER 0.52 mg/g	[76]
Drying 40 °C, 22 h	CO_2_ with 10% Ethanol	13 g/100 L	200 bar; 210 min; 60 °C	TPC 867–1468 mg GAE/kg extract (arbequina), 1487–2073 mg GAE/kg extract (coratina); ABTS 0.607–1.048 mg TRE/g extract (arbequina), 0.807–1.224 mg TRE/g extract (coratina); ORAC 180–318 mg TRE/g extract (arbequina), 215–257 mg TRE/g extract (coratina)	[21]
**Pressurized Liquid Extraction (PLE)**					
Fat removal with N-hexane 1500 psi (room temperature)	50% Ethanol	8 g/11 mL extraction cells	1500 psi; 20 min; 120 °C	Extraction yield 5.8%	[45]
Freeze-dried and fat removal with N-hexane 1500 psi (room temperature)	52.3% Ethanol	5 g/34 mL extraction cells	1500 psi; 20 min; 136.5 °C	Phenols 1659 mg/kg DW (20 compounds); HYT 67 mg/kg DW; OLE 94 mg/kg DW; API 29.8 mg/kg DW; LUT 221 mg/kg DW	[22]
Fat removal with N-hexane 1500 psi (room temperature)	50% Ethanol	8 g/33 mL extraction cells,	1500 psi; 20 min; 120 °C	Freeze-dried extract HYT 2620 mg/kg extract; Oxide of HYT 11,041 mg/kg extract; Acetate of HYT 10,405 mg/kg extract	[53]
Freeze-dried and defatting with CO_2_ flow rate was set at 10.5 kg CO_2_/h for an extraction time of 3 h (20 °C and 6 Mpa)	90% Ethanol	0.8 g/mL	750 rpm/10 Mpa; 20 min; 184 °C	TPC 340 mg GAE/g DE; HYT 9.5 mg/g DE; TYR 5.3 mg/g DE	[24]
Drying 40 °C, 22 h and milled	75% Ethanol	13 g/10 mL	10 MPa; 10 min; 120 °C	TPC 68–72 mg GAE/g extract; ABTS 1116 µmol TRE/g extract (TEAC)	[61]
**High hydrostatic pressure extraction (HHPAE)**					
Drying 40 °C, 24 h	40% Methanol	1 g/10 mL	650 MPa; 1 min; 25 °C	TPC 10.1 g GAE/kg DW, 89.8 g GAE/kg DE TFC 0.1 g/kg DW, 0.6 g/kg DE; HYT 0.9 g/kg DW, 7.8 g/kg DE; LUT ≥ 0.1 g/kg DW, 0.2 g/kg DE	[68]
**Ohmic heating assisted extraction (OHE)**					
Drying 60 °C, 48 h	50% Ethanol	2 g/20 mL	Sinusoidal wave at a frequency of 25 kHz/Electric field 4 V/cm; 30 min; 83 °C	TPC 17.67 mg GAE/g DW; DPPH 3.82 µmol TRE/g DW (IC50); FRAP 150.16 µmol FE(II)/g DW; HYT 33.36 mg/100 g DW; TYR 21.09 mg/100 g DW; OLE 254.38 mg/100 g DW; CAFF 2.59 mg/100 g DW; FER 13.83 mg/100 g DW; API 43.13 mg/100 g DW; RUT 31.46 mg/100 g DW	[30]
**High Pressure-High Temperature Extraction (HPHT)**					
Dried and milled	Methanol	3 g/30 mL	25 bar Atm. of nitrogen; 90 min; 180 °C	TPC 45.2 mg CAE/g DW; TFC 15 mg CE/g DW; HYT 47.5 mg/100 g DW; TYR 236 mg/100 g DW; OLE 1406 mg/100 g DW; CAFF 10.9 mg/100 g DW; API 41.7 mg/100 g DW	[25]
-	50% Ethanol	1 g/10 mL	200 rpm/Max. 2 MPa Atm. of nitrogen; 90 min; 180 °C	TPC 57.7 mg CAE/g DW; DPPH 10.6 mg DPPH/mL extract	[77]
**Pulsed Electric Fields Assisted Extraction (PEF)**					
-	60% Methanol	1 g/10 mL	5000 pulses; 60 min; 25 °C	TPC 15.7 g GAE/kg DW, 108.2 g GAE/kg DE; TFC 0.3 g/kg DW, 2.4 g/kg DE; HYT 2.0 g/kg DW, 13.6 g/kg DE; LUT 0.2 g/kg DW, 1.3 g/kg DE	[68]
**Extraction with deep eutectic solvent (DES)**					
Drying 35 °C, 24 h. and milled	Choline chloride: Citric acid 1:2 molar ratio (20% water)	2 g/25 mL	Homogenization 12,000 rpm; 30 min; 60 °C	TPC 34.08 mg GAE/g DW; DPPH 5.11 g DW/g DPPH (IC50); HYT 3.37 mg/g DW; OLE 12.86 mg/g DW; CAFF 0.01 mg/g DW; LUT 0.12 mg/g DW; RUT 1.71 mg/g DW	[20]
Two-phase centrifugation system. Treated with hydrothermal treatments, sample/water of 1:5 (*w*/*v*) 180 °C; 1.2 Mpa	Choline chloride: glycolic acid: oxalic acid 1:1.7:0.3 molar ratio (without water)	1 g/1 mL	60 min; 120 °C (autoclave)	TPC 28.69 mg GAE/g DW; HYT 85.81 mg/g DW	[50]
-	Citric acid: fructose 1:1 molar ratio (19% water)	10 g/10 g	Stirred; 60 min; 25 °C	Phenols: 3988.74 mg/kg FW (sum of: 3,4 dihydroxyphenyl glycol (3,4-DHPG), hydroxytyrosol glucoside, HYT, tyrosol glucoside (salidroside), VERB, dialdehydic form of decarboxymethyl oleuropein aglycon (3,4-DHPEA-EDA), caffeoil-6′-secologanoside, and comselogoside)	[57]
Drying 35 °C 48 h.	Lactic acid: glucose 5:1 molar ratio (68% water)	0.014 g/mL	210 rpm; 30 min; 80 °C	TPC 15.56 mg GAE/g DW; DPPH 72.75 µmol TRE/g DW; FRAP 178.14 µmol FSE/g DW; HYT 1.24 mg/g DW	[78]
-	Lactic acid: Glucose 5:1 molar ratio (25% water)	1 g FW/25 mL	Magnetic stirring; 60 min; Room temperature	TPC 1349.56 mg GAE/100 g FW; TPF 605.06 mg CE/100 g FW; FRAP 10,603.37 µmol FSE/100 g FW	[58]
	MAE: Choline chloride: lactic acid 1: 2 molar ratio (20% water)	2 g/25 mL	200 W; 30 min; 60 °C	TPC 29.57 mg GAE/g DW; DPPH 17.51 g DW/g DPPH; HYT 0.89 mg/g DW; OLE 7.56 mg/g DW; CAFF 0.01 mg/g DW; LUT 0.17 mg/g DW; RUT 0.74 mg/g DW	[20]
Drying 35 °C, 24 h and milled	UAE: Choline chloride: Citric acid 1:2 molar ratio (20% water)	2 g/25 mL	280 W/60 kHz; 30 min; 60 °C	TPC 20.14 mg GAE/g DW; DPPH 20.69 g DW/g DPPH (IC50); HYT 1.05 mg/g DW; OLE 0.85 mg/g DW; CAFF 0.01 mg/g DW; LUT 0.1 mg/g DW; RUT 0.4 mg/g DW	[20]
Freeze-dried	UAE: Lactic acid: glucose 5:1 molar ratio (15% water)	75 mg/mL	200 W/20 kHz (previously homogenized in vortex 15 s.); 60 min; 40 °C	HYT 111.053 µg/g DW; TYR 1.356 µg/g DW; CAFF 3.225 µg/g DW; FER 8.161 µg/g DW; API 79.328 µg/g DW; LUT 453.690 µg/g DW; RUT 12.620 µg/g DW	[47]
Freeze-dried	UAE: Lactic acid: glucose: water 5:1:9.3 molar ratio	75 mg/mL	200 W/20 kHz (previously homogenized in vortex 15 s.); 60 min; 40 °C	VarietyArauco TPC ≈ 220 µg GAE/mL; HYT 3000 µg/g; LUT 324 µg/g	[56]
Freeze-dried, milled and sieved	UAE: Ammonium acetate: lactic acid 1:7 molar ratio and 1.8% β-cyclodextrins (*w*/*w*) (35% water)	1 g/48 mL	80 W/37 kHz (bath); 22 min; 43 °C	TPC 56.98 mg GAE/g DW	[59]
Drying 35 °C, 24 h and milled	HHPAE: Choline chloride: lactic acid 1: 2 molar ratio (20% water)	2 g/25 mL solvent	600 Mpa; 10 min; Room temperature	TPC 25.96 mg GAE/g DW; DPPH 15.67 g DW/g DPPH (IC50); HYT 2.57 mg/g DW; OLE 1.94 mg/g DW; CAFF 0.01 mg/g DW; LUT 0.12 mg/g DW; RUT 0.66 mg/g DW	[20]

* Average replication. Note: When not explicitly reported, analysis temperature was assumed to be room temperature. SLE: solid–liquid extraction; LLE: liquid–liquid extraction; MAE: microwave-assisted extraction; UAE: ultrasound-assisted extraction; HHPAE: high hydrostatic pressure-assisted extraction; OHE: ohmic heating extraction; DES: deep eutectic solvents; OP: olive pomace; TPC: total phenolic content; TFC: total flavonoid content; HYT: Hydroxytyrosol; TYR: tyrosol; OLE: Oleuropein; CAFF: caffeic acid; FER: ferulic acid; API: apigenin; LUT: luteolin; RUT: rutin; VERB: verbascoside; GAE: gallic acid equivalent; CAE: caffeic acid equivalent; AAE: ascorbic acid equivalent; TRE: Trolox equivalent; FSE: ferrous sulfate equivalents; RE: rutin equivalent; CE: catechin equivalent; DW: dry weight of olive pomace; FW: fresh weight of olive pomace; DE: dry weight extract; DLLME: dispersive liquid–liquid microextraction; TEAC: Trolox equivalent antioxidant capacity; IC50: half-inhibitory concentration; CUPRAC: Cupric Ion Reducing Antioxidant Capacity; MMM: Multi-frequency Multimode Modulated.

## Data Availability

Data presented in this study is available on request from the corresponding authors.

## Supplementary Materials

The following supporting information can be downloaded at https://www.mdpi.com/2304-8158/15/16/2943/s1. Figure S1: Historical evolution of TPC, HPLC, and complementary analytical methodologies employed in OP studies; Figure S2: Schematic representation of green extraction technologies applied for phenolic compound recovery from OP; Table S1: Methods for determining the total phenol content in OP extracts; Table S2: Validation parameters of chromatographic methods used for the determination of phenolic compounds in OP extracts; Table S3: Methods for determining the antioxidant activity in OP extracts; Table S4: Advantages and limitations of the main antioxidant activity assays used for the characterization of OP extracts.

## Abbreviations

The following abbreviations are used in this manuscript:

AAPH	2,2′-azobis(2-amidinopropane) dihydrochloride
ABAP	2,2′-azobis(2-amidinopropane) hydrochloride
ABTS	2,2′-azinobis (3-ethylbenzothiazoline-6-sulfonic acid)
AIBN	α,α-azobis(isobutyronitrile)
AMVN	2,2′-azobis(2,4-dimethylvaleronitrile)
CAE	Caffeic acid equivalents
CAFF	Caffeic acid
CE	Catechin equivalents
CUPRAC	Cupric Ion Reducing Antioxidant Capacity
DAD	Diode array detection
DE	Dry weight extract
DES	Deep eutectic solvents
DLME	Dispersive liquid–liquid microextraction
DPPH	2,2-diphenyl-1-picrylhydrazyl
DW	Dry weight
F-C	Folin–Ciocalteu
Fe(II)	Ferrous ion equivalent
FER	Ferulic acid
FRAP	Ferric Reducing Antioxidant Power
FSE	Ferrous sulfate equivalents
FW	Fresh weight
GAE	Gallic acid equivalents
GAL	Gallic acid
HHPAE	High-hydrostatic pressure-assisted extraction
HPHT	High-pressure and high-temperature extraction
HPLC	High-performance liquid chromatography
HYT	Hydroxytyrosol
IC50	Half-inhibitory concentration
IT	Ion trap
LLE	Liquid–liquid extraction
LUT	Luteolin
*m*/*z*	Mass-to-charge ratio
MAE	Microwave-assisted extraction
MMM	Multi-frequency Multimode Modulated
MS	Mass spectrometry
NADES	Natural deep eutectic solvents
OHE	Ohmic heating extraction
OLE	Oleuropein
OP	Olive pomace
ORAC	Oxygen Radical Absorbance Capacity
PEF	Pulsed electric field-assisted extraction
PLE	Pressurized liquid extraction
PY	Percentage yields
Q	Quadrupole
QE	Quercetin equivalents
QTOF	Quadrupole time-of-flight
QUER	Quercetin
RP-C18	reversed-phase C18 column
RUT	Rutin
SCW	Subcritical water
SE	Steam explosion
SFE	Supercritical fluid extraction
SLE	Solid–liquid extraction
TEAC	Trolox equivalent antioxidant capacity
TOF	Time-of-flight
TPC	Total phenolic content
TPTZ	2,4,6-tris(2-pyridyl)-s-triazine
TQ	Triple quadrupole
TRE	Trolox equivalents
TYR	Tyrosol
UAE	Ultrasound-assisted extraction
UV-Vis	UV-Visible
VAN	Vanillic acid
VERB	Verbascoside
