# Supplementary material for: Valorization of Olive Pomace as a Source of Phenolic Compounds: Extraction Technologies, Analytical Characterization, Biological Activities, and Food Applications
Source: Foods. 2026 Aug 21;15(16):2943. doi: 10.3390/foods15162943 (PMC13512754; doi:10.3390/foods15162943)
Supplement: Supplementary file 1 [file foods-15-02943-s001.zip › foods-4500521-supplementary.pdf]

Review

Valorization of Olive Pomace as a Source of Phenolic Compounds: Extraction Technologies, Analytical Characterization, Biological Activities, and Food Applications

Table S1. Methods for determining the total phenol content in OP extracts.

Sample Extract Volume	F-C Reagent	Reaction Time	Sodium Carbonate Reagent	Wavelength	Time Incubated	Temperature	Standar	Ref
**100-300 µL**	625 µL	3 min	2.5 mL (35% w/v)	725	1 h	Room	CAE	[40]
**200 µL**	1500 µL	5 min	1500 µL (7% w/v)	725	1 h	Room	GAE	[44]
**20 µL (and 1.58 mL water)**	100 µL	8 min	300 µL (20% w/v)	765	2 h	Room	QE	[46]
**100 µL (and 7.9 mL water)**	500 µL	n/s	1.5 mL (supersaturated)	765	2 h	Room	GAE	[20]
**1000 µL (and 7.5 mL water)**	500 µL	4 min	1000 µL (5% w/v)	725	1 h	Room	GAE	[75]
**30 µL**	150 µL	n/s	120 µL (7.5% w/v)	765	15 min (45°C) and after 30 min room temperature.	Room	GAE	[49]
**100 µL (and 1.6 mL water)**	100 µL	5 min	200 µL	765	2 h	Room	GAE	[76]
**5 µL**	250 µL	n/s	200 µL (20% w/v)	655	10 min	Room	GAE	[50]
**300 µL**	3000 µL	n/s	2 mL (10% w/v)	760	1 h	Room	GAE	[51,52]
**40 µL (and 3 mL water)**	200 µL	5 min	600 µL (20% w/v)	765	30 min	40°C	GAE	[24]
**20 µL (and 1.6 mL water)**	100 µL	n/s	300 µL (20% w/v)	765	30 min	40 °C	GAE	[54]
**10 µL**	50 µL	2 min	200 µL	750	30 min	Room	GAE	[21]
**50 µL (and 3.5 mL water)**	200 µL	5 min	1250 µL (5% w/v)	750	60 min	Room	GAE	[56]
**.**	1250 µL	1 min	2.5 mL	760	5 min	50°C	GAE	[73]
**100 µL (and 7.9 mL water)**	500 µL	n/s	1500 µL (sat.)	765	2 h	Room	GAE	[68]
**200 µL**	1000 µL	n/s	800 µL (7.5% w/v)	765	30 min	Room	GAE	[69]
**20 µL (and 1.580 mL water)**	100 µL	8 min	300 µL (20% w/v)	765	2 h	Room	GAE	[59]
**70 µL (and 3.9 mL water)**	250 µL	n/s	750 µL (7.5% w/v)	765	n/s	Room	GAE	[58]

Note: When not explicitly reported, analysis temperature was assumed to be room temperature n/s: not specified; F-C: Folin-Ciocalteu; CAE: caffeic acid equivalent; QE: quercetin equivalent; GAE: gallic acid equivalent.

Table S2. Validation parameters of chromatographic methods used for the determination of phenolic compounds in OP extracts.

Compound	Instrument	LOD (μg/g)	LOQ (μg/g)	Repeatability (RSD %)	Reproducibility (RSD%)	Recovery (%)	Reference
**HYT**	HPLC-DAD	0.023	0.077	2.40	3.57	97.99	[47]
**TYR**	C18 (150 mm ×	0.089	0.297	1.00	2.68	109.37	
**CAFF**	4.6 mm; 5 μm)	0.013	0.043	7.03	9.68	101.05	
**RUT**		0.003	0.010	8.61	10.37	100.04	
**FER**		0.010	0.034	1.55	3.15	109.63	
**LUT**		0.013	0.043	0.35	1.59	89.79	
**API**		0.009	0.029	1.45	2.68	92.57	
**HYT**	HPLC- UV	0.43	1.30	1.03	-	103	[56]
**LUT**	C18 (100 mm × 3.0 mm; 2.6 μm)	0.05	0.15	1.97	-	89	

HYT, hydroxytyrosol; TYR, tyrosol; CAFF, caffeic acid; RUT, rutin; FER, ferulic acid; LUT, luteolin; API, apigenin; LOD: limit of detection; LOQ: limit of quantification; RSD: relative standard deviation.

Table S3. Methods for determining the antioxidant activity in OP extracts.

Method	Reactive Volume	Sample	Time	Wavelength	Ref
**ABTS**	187 μL ABTS*, diluted with 80% ethanol.	13 μL	30 min	414-655-750	[42]
	3 mL (7 mM) ABTS*, diluted with phosphate buffer (pH 7.4).	30 μL	6 min	734	[51,52]
	3 mL (7 mM) ABTS*, diluted with water.	30 μL	6 min	734	[54]
	190 μL (7mM) ABTS*, diluted with ethanol.	10 μL	10 min	750	[21]
**DPPH**	2.7 mL (60 μM of DPPH solution in methanol)	0.3 mL	1 h	517	[44]
	1.5 mL (20 mg/dL; ~ 0.51 mM of DPPH solution in methanol)	0.75 mL	15 min	517	[46]
	3.9 mL (0.0025 g/ 100 mL; ~63.4 μM of DPPH solution in methanol)	0.1 mL	20 min	515	[20,68]
	270 μL (60 μM of DPPH solution in ethanol)	30 μL	30 min	515	[49]
	2 mL of DPPH solution in methanol	200 μL	15 min	517	[51,52]
	2 mL (0.2 mM of DPPH solution in methanol)	1 mL	n/s	517	[54]
	3500 μL (0.045 mg/mL; 114 μM of DPPH solution in methanol)	250 μL	30 min	515	[56]
	2.7 mL (60 μM of DPPH solution in 80% (v/v) methanol)	0.3 mL	30 min	517	[78]
	900 μL (60 μM of DPPH solution in ethanol)	100 μL	n/s	517	[69]
	1980 μL (40 μg/m of DPPH solution in methanol)	20 μL	30 min	517	[60]
**FRAP**	265 μL of FRAP solution	35 μL	30 min (37°C)	595	[49]
	3 mL of FRAP solution	100 μL	6 min	593	[51,52]
	300 mL of FRAP solution	n/s	5 min	593	[73]
	265 μL of FRAP solution	35 μL	n/s	595	[58]

n/s: not specified; ABTS: 2,2′- azino-bis 3-ethylbenzothiazoline-6-sulfonic acid; DPPH: (2,2-diphenyl-1-picrylhydrazyl); FRAP: Ferric Reduction of Antioxidant Power; AAPH: 2,2-azobis (2-amidino-propane) dihydrochloride; *: ABTS with potassium persulfate until an absorbance of 0.7.

Table S4: Advantages and limitations of the main antioxidant activity assays used for the characterization of OP extracts.

Assay	Limitations	Advantages
**ABTS**	Colored matrices may interfere with the analysis. Limited biological relevance. The reaction may proceed slowly, potentially leading to underestimated results when short analysis times are used. Susceptible to interference from other compounds.	Applicable to a wide range of antioxidant compounds and sample matrices. Simple, rapid, and relatively inexpensive.
**DPPH**	Interference from colored matrices. Suitable for organic or hydroalcoholic systems but limited for highly aqueous samples. Results may be affected by reaction time and temperature.	Low cost, easy implementation, good reproducibility, suitable for room-temperature analysis, and amenable to automation.
**FRAP**	Strongly dependent on reaction time. Several modifications of the method have been proposed, making comparisons between studies more difficult. Mainly applicable to hydrophilic matrices. Measures reducing power rather than direct radical scavenging activity.	Simple, rapid, and inexpensive. High sensitivity and precision.
**CUPRAC**	Measures reducing capacity, and may be affected by interfering compounds. Does not fully reflect physiological conditions.	Rapid assay that is not strongly limited by reaction time. Stable and accessible reagents. Applicable to a wide variety of matrices. Limited influence of environmental factors such as light, air, and humidity. Wide linear range and broad applicability.
**ORAC**	Affected by temperature variations. Requires fluorescence instrumentation and longer analysis times than other antioxidant assays.	High biological relevance because it evaluates radical scavenging against peroxyl radicals under conditions closer to biological systems. Applicable to different sample matrices.

ABTS, 2,2′-azino-bis(3-ethylbenzothiazoline-6-sulfonic acid); DPPH, 2,2-diphenyl-1-picrylhydrazyl; FRAP, ferric reducing antioxidant power; CUPRAC, cupric ion reducing antioxidant capacity; ORAC, oxygen radical absorbance capacity. Source: Compiled by the authors based on data reported by [31].

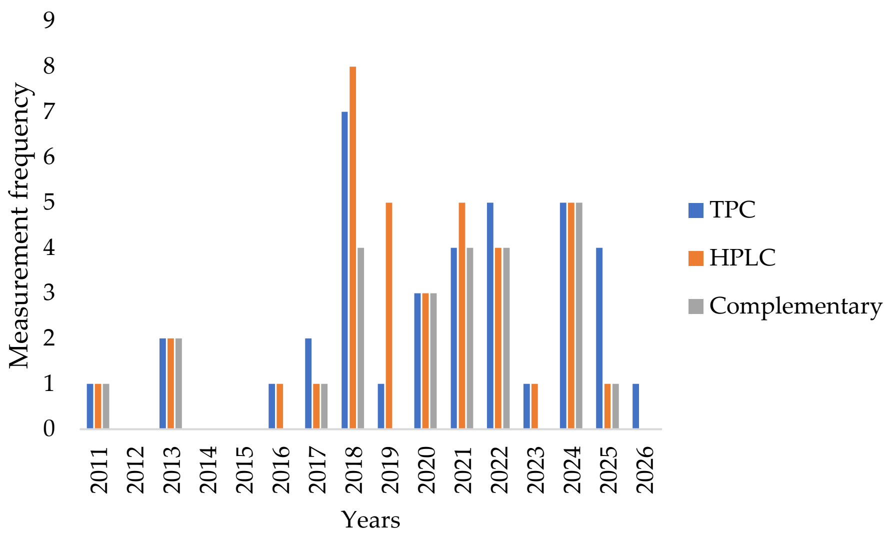

Figure S1: Historical evolution of TPC, HPLC, and complementary analytical methodologies employed in OP studies.

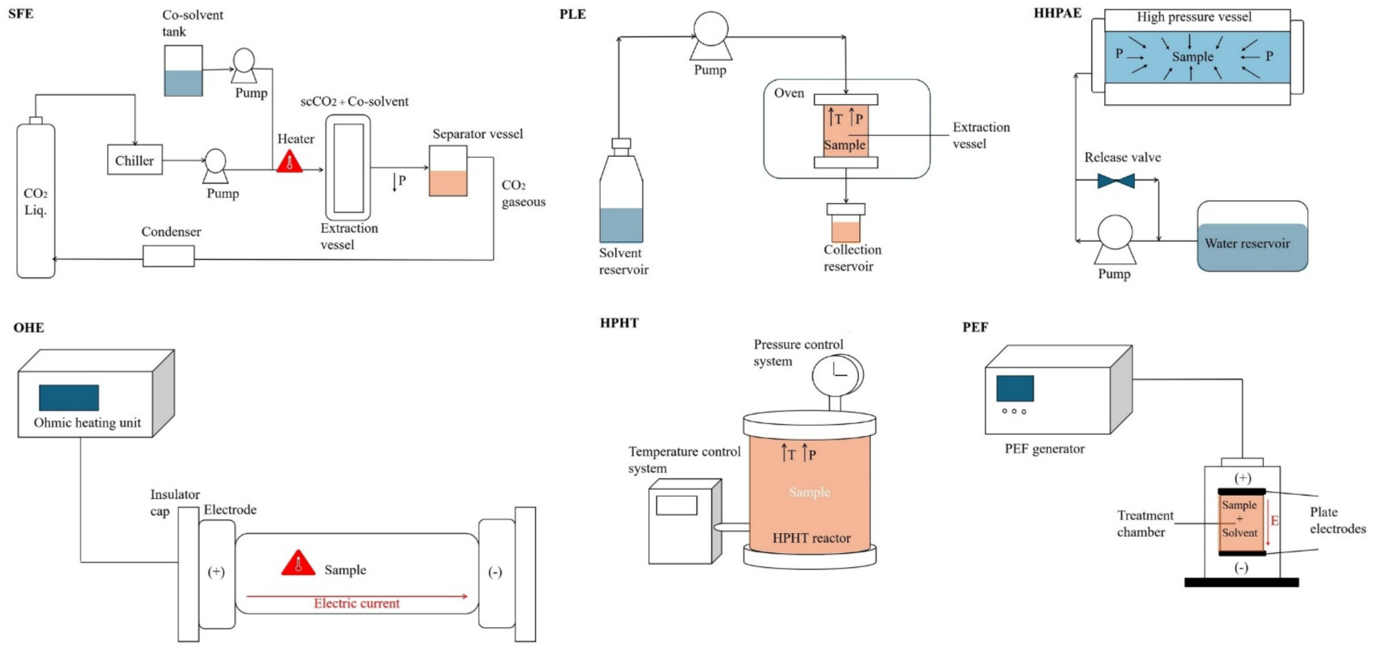

Figure S2. Schematic representation of green extraction technologies applied for phenolic compound recovery from OP (SFE, PLE and PEF adapted from [106]). SFE: supercritical fluid extraction; PLE: pressurized liquid extraction; HHPAE: high-hydrostatic pressure-assisted extraction; OHE: ohmic heating extraction; HPHT: high-pressure and high-temperature extraction; PEF: pulsed electric field-assisted extraction; P: pressure; T: temperature; E: electric field.

References:

20. Chanioti, S.; Tzia, C. Extraction of Phenolic Compounds from Olive Pomace by Using Natural Deep Eutectic Solvents and Innovative Extraction Techniques. *Innov. Food Sci. Emerg. Technol.* **2018**, *48*, 228–239. https://doi.org/10.1016/j.ifset.2018.07.00.

21. Dauber, C.; Carreras, T.; Fernández Fernández, A.; Irigaray, B.; Albores, S.; Gámbaro, A.; Ibáñez, E.; Vieitez, I. Response Surface Methodology for the Optimization of Biophenols Recovery from "Alperujo" Using Supercritical Fluid Extraction. Comparison between Arbequina and Coratina Cultivars. *J. Supercrit. Fluids* **2022**, *180*, 105460. https://doi.org/10.1016/j.supflu.2021.105460.

24. Katsinas, N.; Bento Da Silva, A.; Enríquez-de-Salamanca, A.; Fernández, N.; Bronze, M.R.; Rodríguez-Rojo, S. Pressurized Liquid Extraction Optimization from Supercritical Defatted Olive Pomace: A Green and Selective Phenolic Extraction Process. *ACS Sustain. Chem. Eng.* **2021**, *9*, 5590–5602. https://doi.org/10.1021/acssuschemeng.0c09426.

40. Lafka, T.-I.; Lazou, A.E.; Sinanoglou, V.J.; Lazos, E.S. Phenolic and Antioxidant Potential of Olive Oil Mill Wastes. *Food Chem.* **2011**, *125*, 92–98. https://doi.org/10.1016/j.foodchem.2010.08.041.

42. Rubio-Senent, F.; Rodríguez-Gutíerrez, G.; Lama-Muñoz, A.; Fernández-Bolaños, J. New Phenolic Compounds Hydrothermally Extracted from the Olive Oil Byproduct Alperujo and Their Antioxidative Activities. *J. Agric. Food Chem.* **2012**, *60*, 1175–1186. https://doi.org/10.1021/jf204223w.

44. Ramos, P.; Santos, S.A.O.; Guerra, Â.R.; Guerreiro, O.; Felício, L.; Jerónimo, E.; Silvestre, A.J.D.; Neto, C.P.; Duarte, M. Valorization of Olive Mill Residues: Antioxidant and Breast Cancer Antiproliferative Activities of Hydroxytyrosol-Rich Extracts Derived from Olive Oil by-Products. *Ind. Crops Prod.* **2013**, *46*, 359–368. https://doi.org/10.1016/j.indcrop.2013.02.020.

46. Zagmutt, S.; Guzmán, L.; Orrego, R.; Wehinger, S.; Leiva, E. Phenolic Compound Identification and Antioxidant Capacity of Alperujo Extracts from Region Del Maule, Chile. *Int. J. Food Prop.* **2016**, *19*, 2016–2025. https://doi.org/10.1080/10942912.2015.1092162.

47. Fernández, M.D.L.Á.; Espino, M.; Gomez, F.J.V.; Silva, M.F. Novel Approaches Mediated by Tailor-Made Green Solvents for the Extraction of Phenolic Compounds from Agro-Food Industrial by-Products. *Food Chem.* **2018**, *239*, 671–678. https://doi.org/10.1016/j.foodchem.2017.06.150.

49. Nunes, M.A.; Costa, A.S.G.; Bessada, S.; Santos, J.; Puga, H.; Alves, R.C.; Freitas, V.; Prior Pinto Oliveira M.B. Olive Pomace as a Valuable Source of Bioactive Compounds: A Study Regarding Its Lipid- and Water-Soluble Components. *Sci. Total Environ.* **2018**, *644*, 229–236. https://doi.org/10.1016/j.scitotenv.2018.06.350.

50. Fernández-Prior, M.Á.; Charfi, A.; Bermúdez-Oria, A.; Rodríguez-Juan, E.; Fernández-Bolaños, J.; Rodríguez-Gutiérrez, G. Deep Eutectic Solvents Improve the Biorefinery of Alperujo by Extraction of Bioactive Molecules in Combination with Industrial Thermal Treatments. *Food Bioprod. Process.* **2020**, *121*, 131–142. https://doi.org/10.1016/j.fbp.2020.02.001.

51. Gómez-Cruz, I.; Contreras, M.D.M.; Carvalheiro, F.; Duarte, L.C.; Roseiro, L.B.; Romero, I.; Castro, E. Recovery of Bioactive Compounds from Industrial Exhausted Olive Pomace through Ultrasound-Assisted Extraction. *Biology* **2021**, *10*, 514. https://doi.org/10.3390/biology10060514.

52. Gómez-Cruz, I.; Contreras, M.D.M.; Romero, I.; Castro, E. Optimization of Microwave-Assisted Water Extraction to Obtain High Value-Added Compounds from Exhausted Olive Pomace in a Biorefinery Context. *Foods* **2022**, *11*, 2002. https://doi.org/10.3390/foods11142002.

54. Macedo, G.A.; Santana, Á.L.; Crawford, L.M.; Wang, S.C.; Dias, F.F.G.; De Moura Bell, J.M.L.N. Integrated Microwave- and Enzyme-Assisted Extraction of Phenolic Compounds from Olive Pomace. *LWT* **2021**, *138*, 110621. https://doi.org/10.1016/j.lwt.2020.110621.

56. Morgana, N.M.; Magdalena, E.; Fernandez, M.D.L.A.; Fernanda, S.M. NADES for Food Industry Innovation: Novel Bioadditives Based on Olive Oil Byproducts. *Food Bioprod. Process.* **2022**, *134*, 193–201. https://doi.org/10.1016/j.fbp.2022.05.007.

58. Soares, T.F.; Páscoa, R.N.M.J.; Fonseca, J.; Souto, E.B.; Prior Pinto Oliveira, M.B.; Alves, R.C. Development and Characterization of Natural Deep Eutectic Solvents for Optimized Extraction of Olive Pomace Phenolics. *Food Bioprod. Process.* **2025**, *154*, 295–306. https://doi.org/10.1016/j.fbp.2025.10.002.
59. Pietrangeli, G.; Della Posta, S.; D'Alessandro, E.; Giannitelli, S.M.; Trombetta, M.; Maggi, C.; De Gara, L.; Fanali, C. Sustainable Supramolecular Deep Eutectic Solvents Based Extraction for Bioactive Phenolic Compounds Recovery from Olive Pomace. *Adv. Sample Prep.* **2025**, *13*, 100166. https://doi.org/10.1016/j.sampre.2025.100166.
60. Expósito-Almellón, X.; Munguía-Ubierna, Á.; Duque-Soto, C.; Borrás-Linares, I.; Quirantes-Piné, R.; Lozano-Sánchez, J. Optimized Ultrasound-Assisted Extraction for Enhanced Recovery of Valuable Phenolic Compounds from Olive By-Products. *Antioxidants* **2025**, *14*, 938. https://doi.org/10.3390/antiox14080938.
68. Tsevdou, M.; Ntzimani, A.; Katsouli, M.; Dimopoulos, G.; Tsimogiannis, D.; Taoukis, P. Comparative Study of Microwave, Pulsed Electric Fields, and High Pressure Processing on the Extraction of Antioxidants from Olive Pomace. *Molecules* **2024**, *29*, 2303. https://doi.org/10.3390/molecules29102303.
69. Guerboub, L.; Soufi-Maddi, O.; Ouldsaadi, L.; Bachir Bey, M.; Medouni-Haroune, L.; Madani, K.; Boulekbache-Makhlouf, L. Ultra-Turrax Homogenization–Assisted Extraction of Bioactive Compounds from Olive Pomace Using Response Surface Methodology. *Food Anal. Methods* **2025**, *18*, 2198–2207. https://doi.org/10.1007/s12161-025-02861-4.
73. Marđokić, A.; Maldonado, A.E.; Klosz, K.; Molnár, M.A.; Vatai, G.; Bánvölgyi, S. Optimization of Conditions for Microwave-Assisted Extraction of Polyphenols from Olive Pomace of Žutica Variety: Waste Valorization Approach. *Antioxidants* **2023**, *12*, 1175. https://doi.org/10.3390/antiox12061175.
75. Mojerlou, Z.; Elhamirad, A. Optimization of Ultrasound-Assisted Extraction (UAE) of Phenolic Compounds from Olive Cake. *J. Food Sci. Technol.* **2018**, *55*, 977–984. https://doi.org/10.1007/s13197-017-3005-x.
76. Caballero, A.S.; Romero-García, J.M.; Castro, E.; Cardona, C.A. Supercritical Fluid Extraction for Enhancing Polyphenolic Compounds Production from Olive Waste Extracts. *J. Chem. Technol. Biotechnol.* **2020**, *95*, 356–362. https://doi.org/10.1002/jctb.5907.
78. Cabrera, L.; Xavier, L.; Zecchi, B. Extraction of Phenolic Compounds with Antioxidant Activity from Olive Pomace Using Natural Deep Eutectic Solvents: Modelling and Optimization by Response Surface Methodology. *Discov. Food* **2024**, *4*, 29. https://doi.org/10.1007/s44187-024-00100-z.
106. Sezer Okur, P.; Okur, I. Recent Advances in the Extraction of Phenolic Compounds from Food Wastes by Emerging Technologies. *Food Bioprocess Technol.* **2024**, *17*, 4383–4404. https://doi.org/10.1007/s11947-024-03365-5
